# Concurrent TMS-EEG to characterise interhemispheric signal propagation over the motor and prefrontal regions: An independent replication of GABA_B_ receptor mechanisms

**DOI:** 10.1371/journal.pone.0360250

**Published:** 2026-10-16

**Authors:** Jie Zhou, Yiting He, Chenxia Jin, Chen Yang, Yazhen Han, Yujun Wang, Zhen Zhen, Zhimin Huang, Shuyan Shi, Xianwei Che, Jiqing He

**Affiliations:** 1 Centre for Cognition and Brain Disorders, The Affiliated Hospital of Hangzhou Normal University, Hangzhou, China; 2 Department of Obstetrics, The Affiliated Hospital of Hangzhou Normal University, Hangzhou, China; Beijing Sport University, CHINA

## Abstract

Callosal abnormalities disrupt interhemispheric communications that impair the recovery of a series of neurological and psychiatric disorders. Understanding interhemispheric communication could help identify treatments for disorders with dysfunctional callosal transmission. This study aimed to validate the sensitivity of a TMS-EEG-based measure of interhemispheric signal propagation (ISP) to GABA_B_ receptor modulation, while independently replicating the pharmacological mechanisms of interhemispheric inhibition. A group of 29 participants received an oral dose of either baclofen, a GABA_B_ receptor agonist, or a placebo. Before and after drug intake, single-pulse TMS-EEG was applied to assess ISP in both the motor and prefrontal regions. Our findings indicated that baclofen increased interhemispheric inhibition over the motor region, indexed by significantly decreased ISP (*p* = 0.037). No significant effect was observed in the dorsolateral prefrontal region (*p* = 0.186), suggesting regional specificity in GABA_B_ receptor modulation of ISP. These findings uncovered the pharmacological mechanisms of interhemispheric inhibition as indexed by ISP. They also highlight the potential of ISP for characterising interhemispheric communication, which could inform the treatment for a series of disorders with dysfunctional callosal transmission.

## Introduction

The corpus callosum, the brain’s largest white matter tract, facilitates interhemispheric communication between homologous regions [1]. Callosal abnormalities disrupt interhemispheric communications, impairing recovery in neurological and psychiatric disorders, including stroke [2], schizophrenia [3], and autism [4]. Understanding interhemispheric communication could help identify treatments for disorders in which callosal transmission is dysfunctional.

Using TMS and electroencephalography co-registration (TMS-EEG), interhemispheric signal propagation (ISP; distinct from the ipsilateral silent period, another measure of transcallosal communication [5,6]) measures the transmission of cortical evoked activity between contralateral hemispheres. Previous studies indicated that ISP correlates with callosal integrity [7], and can also be applied to other cortices beyond the motor region [8–10]. It is important to note that ISP represents one of several approaches to assess transcallosal inhibition. Other established measures include the ipsilateral silent period, which assesses transcallosal inhibition through suppression of ongoing EMG activity during voluntary contraction [6], and dual-coil TMS paradigms, which probe interhemispheric inhibitory circuits using conditioning and test pulses delivered to contralateral motor cortices [5]. More recently, TMS-EEG studies have identified the M1-P15 component as a putative marker of transcallosal inhibition, with shorter P15 latencies reflecting faster interhemispheric conduction [11,12]. However, these measures are restricted to motor regions or require active muscle participation. In contrast, ISP can be applied to both motor and non-motor regions without requiring motor output, thereby extending the assessment of interhemispheric communication to prefrontal and other cortical areas [8–10].One pioneering study identified that ISP has a high reliability across assessments [13]. More importantly, the same study found that the inhibitory effects observed with interhemispheric signal transmission are mediated by GABA_B_ receptor neurotransmission [13]. These novel findings demonstrate ISP as a valuable tool to characterise interhemispheric connectivity. They also provide insights into the design of non-invasive brain stimulation technologies for disorders with dysfunctional callosal transmission.

Given the critical role of ISP, however, the pharmacological mechanisms have never been independently replicated in another study. Moreover, the previous study relied on a sample size of 12 participants [13], further raising the need to validate these novel findings. The current study was therefore designed to independently replicate the mechanisms of GABA_B_ receptor neurotransmission in interhemispheric communications. A group of 29 participants received an oral dose of either baclofen, a GABA_B_ receptor agonist, or a placebo. Before and after drug intake, single-pulse TMS-EEG was applied to assess ISP in both the motor and prefrontal cortex. The current study was designed with two complementary aims: (1) to conduct an independent replication of GABA_B_ receptor effects on ISP with an expanded sample size; and (2) to validate ISP as a sensitive pharmacodynamic marker for GABAergic modulation across cortical regions. To achieve these aims, we employed a double-blind, placebo-controlled, crossover design to assess ISP changes following baclofen administration in both the motor and prefrontal cortices.

## Materials and methods

### Participants

A group of 29 healthy, right-handed adults who met the eligibility criteria [14] for TMS were recruited for this study. Participants with a history or current diagnosis of psychiatric disorders, or those taking psychoactive medications, were excluded with the Mini International Neuropsychiatric interview (MINI) [15]. This sample size is larger than a previous study (n = 12), which identified that oral baclofen led to a significant decrease in ISP [13]. Participants were recruited from 10 November 2021–1 March 2022. Written informed consent was obtained from all participants before the commencement of this study. Ethical approval was granted by the Ethics Committee of the Affiliated Hospital of Hangzhou Normal University (2021-E2-HS-065), and the study was conducted following the Declaration of Helsinki.

### Experimental design and procedure

This study was a double-blind, placebo-controlled, crossover design with randomized and counterbalanced session order. Each participant attended two sessions, spaced at least seven days apart (Fig 1). Specifically, participants were randomly allocated to one of two sequence orders (baclofen-placebo or placebo-baclofen) following a computer-generated randomization schedule (MATLAB, The MathWorks, USA). This procedure, conducted by an independent researcher not involved in data collection, ensured that potential sequence effects were evenly distributed between conditions [16,17].During each session, they initially received a single-pulse TMS assessment in the primary motor cortex (M1) and the dorsolateral prefrontal cortex (DLPFC) (labelled ‘PRE’). Then, participants were administered an oral dose of the drug (baclofen or placebo), followed by a 90-minute waiting period. A second single-pulse TMS assessment was performed at the end of the waiting period (labelled ‘POST’).

**Fig 1:**
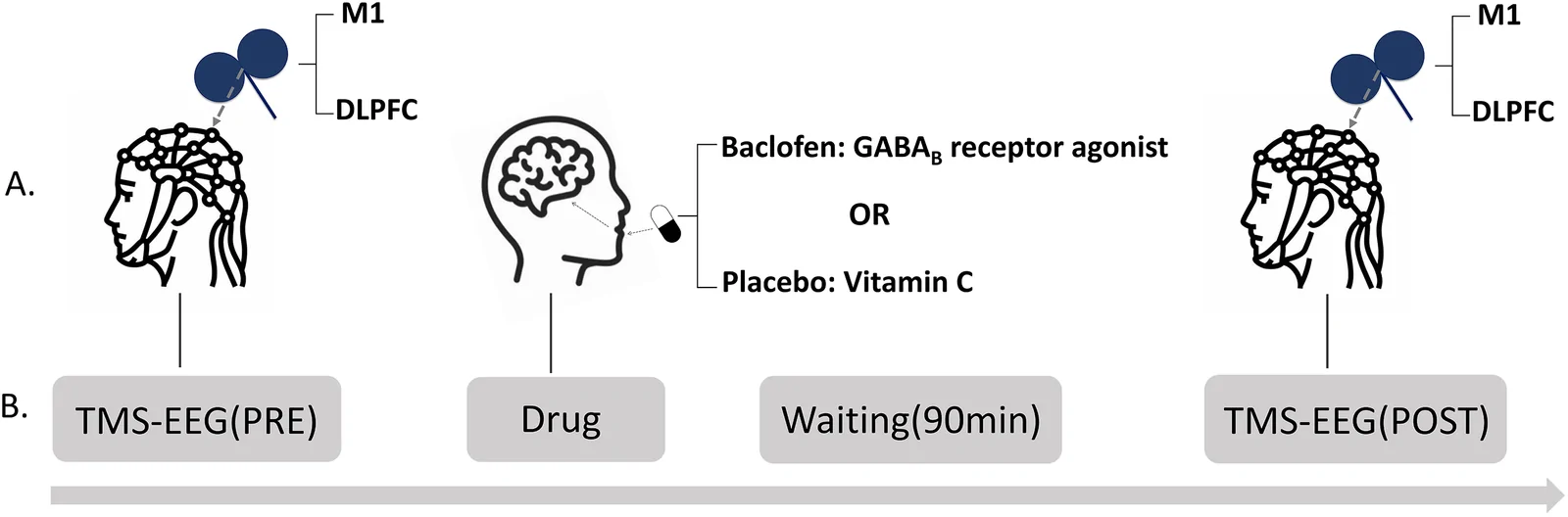
Experimental design and procedure. Each participant attended the laboratory on two separate occasions. In each session, participants received a randomized oral dose of baclofen or a placebo and underwent pre- and post-TMS-EEG assessments (M1 and DLPFC).

### Drug administration and blinding

In each session, participants received a single oral dose of either Baclofen (10 mg, a GABAB receptor agonist) or Vitamin C (100 mg, serving as the placebo), followed by a 90-minute waiting period. The 10 mg dose was selected based on previous studies demonstrating that this dose produces measurable pharmacodynamic effects in healthy volunteers while maintaining good safety and tolerability profiles [18–20].This dose is consistent with prior TMS-EEG studies investigating GABAergic neurotransmission [21,22]. Both drugs were identical in appearance and were stored in identical glass containers labelled differently. Both the experimenter (S.S.) and the participants were unaware of the specific drug in each session, ensuring blinding integrity.

### Resting motor threshold and TMS-EEG

The resting motor threshold (RMT) was initially assessed. Resting motor threshold (RMT), defined as the minimum intensity to induce motor-evoked potentials (MEPs)  > 0.05 mV of the first dorsal interosseous (FDI) muscle in 5/10 trials, was measured before each session. Surface electromyography (EMG) was recorded from the right FDI muscle using disposable Ag/AgCl electrodes in a belly-tendon montage during RMT determination only. During TMS-EEG recordings, EMG was not continuously monitored to avoid electrical interference with EEG signals and to minimize participant discomfort [23]. Single pulses to the hand region of the left M1 (45° to the midline, handle pointing backward) at 4s ± 10% jitter intervals were sent by a figure-eight coil connected to a Magstim Rapid2 system (Magstim Company Ltd, UK). To ensure consistency across procedures, RMT determination was performed while participants wore an EEG cap, as single-pulse TMS was also conducted with the cap on.

Single-pulse TMS was applied to both the left primary motor cortex (M1) and the left dorsolateral prefrontal cortex (DLPFC). For M1 localization, the motor ‘hotspot’ was identified using a semi-grid systematic search procedure. The coil was initially positioned over the estimated hand knob area (approximately 5 cm lateral and 2 cm anterior to the vertex), then adjusted in 0.5-cm steps in an anterior-posterior and medial-lateral grid pattern to locate the site eliciting the largest peak-to-peak motor evoked potential (MEP) amplitude in the first dorsal interosseous (FDI) muscle. The identified hotspot was marked on the EEG cap for consistent repositioning across sessions. The coil was oriented with the handle pointing posterolaterally at a 45° angle relative to the sagittal midline, inducing a posterior-to-anterior (PA) current flow in the underlying cortex [24].For DLPFC localization, the left F3 position was determined using the Beam F3 methodology [25]. This approach measures the distance from nasion to inion and from left to right preauricular points, calculating F3 as the intersection point 50% of the distance from Fpz to FCz and 50% of the distance from F7 to Fz on the left side. The coil was oriented with the handle pointing laterally at a 90° angle relative to the sagittal midline (perpendicular to the midline), inducing a lateral-to-medial (LM) current flow [25,26]. The stimulation intensity was set to 110% of the resting motor threshold (RMT) [24]. A total of 105 single pulses were delivered at each site to enhance the signal-to-noise ratio in TMS-EEG recordings [17,24]. During stimulation, white noise masking was delivered through inserted earphones at an intensity sufficient to render the TMS coil click barely audible [16]. In terms of coil configuration, the induced current flowed from the front of the coil toward the handle with this coil [27].The choice of 110% RMT is, supported by evidence that this suprathreshold intensity elicits robust TEPs while maintaining tolerability [28–31].

EEG data during single-pulse TMS were collected in a temperature-regulated, soundproof, and electrically shielded environment. Participants remained seated in a chair with their eyes open, maintaining a forward gaze. Continuous EEG was recorded using a 64-channel cap with BrainAmp DC amplifiers (Brain Products GmbH, Germany) at a sampling rate of 5,000 Hz. The FCz electrode served as the reference, while AFz was used as the ground. Throughout the recording session, electrode impedances were maintained below 5 kΩ.

### TMS-EEG data analysis

TMS-EEG data was processed using the TMS-EEG Signal Analyzer (TESA) toolbox and a custom script implemented in MATLAB(R2022b, The MathWorks, USA) [23]. Step-by-step, detailed procedures can be found here: https://nigelrogasch.gitbook.io/tesa-user-manual/example_pipelines. The data were initially segmented into epochs around the TMS pulses (−1,000–1,000 ms) and baseline corrected using the −500 to −5 ms window. The large artifacts caused by the magnetic pulses were removed and interpolated between −5 and 25 ms. The −5–25 ms interpolation window was selected based on direct inspection of the TMS-related artifact in this dataset. While the initial amplifier recovery occurred within approximately 5–12 ms, a secondary artifact component arising from cranial muscle activation was observed to persist up to approximately 20–25 ms, with considerable inter-individual variability in its duration. To ensure complete and consistent removal of the full artifact time course for all participants, a conservative fixed window of −5–25 ms was applied. A representative example of the artifact and the interpolation window is provided in S1 Fig. Importantly, as our primary outcome measure (ISP) focuses on the 50–150 ms post-stimulus interval for the stimulated hemisphere and 60–160 ms for the contralateral hemisphere [7,13,32], the interpolation window does not affect the neural signals of interest. Following this the data was downsampled to 1,000 Hz and manually inspected for bad channels and trails. An initial round of FastICA was conducted to eliminate muscle and decay artifacts [33]. The data were then filtered using Butter filters (band-pass = 1–100 Hz; band-stop = 48–52 Hz). On average, 10.0 ± 5.7 components were rejected for the motor cortex under the placebo condition, and 8.9 ± 4.1 under the baclofen condition. For the dorsolateral prefrontal cortex (DLPFC), 14.3 ± 5.5 components were rejected under placebo and 13.4 ± 5.3 under baclofen. The number of components removed in the first ICA round reflects the cumulative total of artifactual components identified across multiple ICA runs, with each step manually inspected to confirm that only artifactual components were removed while neural signals were retained (S2 Fig). A second round of FastICA was applied to remove no-neural artifacts including eye blinks, eyes movements, sustained muscle activity, and electrode noise [33]. For the motor cortex, an average of 25.9 ± 6.2 components were rejected under placebo and 25.0 ± 4.3 under baclofen. For the DLPFC, 19.1 ± 5.2 components were rejected under placebo and 18.4 ± 4.3 under baclofen. Blink, eye movement, muscle, and electrode noise components were identified and removed automatically based on predefined heuristics. Interpolation was then performed for removed channels. Bad channels, if any, were interpolated using spherical spline interpolation. Finally, the data were re-reference to the common average. The number of channels removed and interpolated was recorded for each condition. In the motor cortex, the mean number of interpolated channels was 1.00 ± 1.00 for baclofen and 0.89 ± 0.97 for placebo. In the dorsolateral prefrontal cortex, the mean number of removed channels was 8.74 ± 5.05 for baclofen and 7.52 ± 5.47 for placebo. No significant differences were observed between drug and placebo conditions in any region, indicating consistent data quality across sessions. The number of trials retained after artifact rejection was recorded for each condition. In the motor cortex, the mean number of trials was 97.26 ± 2.84 for baclofen PRE, 97.04 ± 2.03 for baclofen POST, 96.89 ± 4.67 for placebo PRE, and 96.22 ± 2.34 for placebo POST. In the DLPFC, the mean number of trials was 83.33 ± 10.83 for baclofen PRE, 84.67 ± 8.16 for baclofen POST, 81.96 ± 12.97 for placebo PRE, and 81.89 ± 11.94 for placebo POST. To justify the sufficient number of trials, established TMS-EEG literature was referred. For the M1, Lioumis et al. (2009) reported a high reproducibility (r > 0.80) with ~100 pulses per condition and at least 80 epochs retained for averaging [34]. For the DLPFC, Noda et al. (2024) demonstrated that 80 pulses yield TEP waveforms equivalent to 160 or 240 pulses, while 40 pulses are insufficient. In our dataset, the retained trial numbers are sufficient for reliable TEP estimation [35].A representative example of the EEG signal before and after preprocessing is provided in S3 Fig, illustrating the effectiveness of the artifact removal procedure across all conditions.

### TMS-induced ISP

Interhemispheric signal propagation (ISP) was assessed by computing the ratio between TEPs from the unstimulated hemisphere and those from the stimulated hemisphere. In the motor region, ISP was calculated using the averaged-trial rectified TEP curve from the C3 electrode (closest electrode to the left motor hand region) and the C4 electrode (the corresponding electrode over the right motor region) [7,13]. In the dorsolateral prefrontal region, the recording electrodes of interest were F3 (left hemisphere) and F4 (right hemisphere) [13]. For the left (stimulated) hemisphere, the area under the curve was calculated within the 50–150 ms post-stimulus interval [7]. An average interhemispheric transmission time of 10 ms was adopted, as reported in previous studies [5,6]. Accordingly, for the right (unstimulated) hemisphere, a 60–160 ms time window was applied in line with established methodologies [7,32,36]. ISP was calculated using the formula listed below.


$$\begin{array}{c} \text{ISP}\left(\%\right)=\frac{\text{area under rectified curve }\left(\text{non stimulated hemisphere}\right)}{\text{area under rectified curve }\left(\text{stimulated hemisphere}\right)}\times \text{100} \end{array}$$

### Statistical analysis

Statistical analyses were conducted using SPSS 25.0 (SPSS Inc., Chicago, IL, USA). For ISP, a two-way repeated-measures analysis of variance (rmANOVA) was performed on raw ISP values with drug condition (baclofen and placebo) and time point (PRE and POST) as within-subject factors. The critical test for drug effects was the Drug × Time interaction. For post-hoc comparisons, change scores (ΔISP = ISP_POST_ − ISP_PRE_) were calculated and compared between drug conditions using paired t-tests with Bonferroni correction (*p* < 0.05). These change scores were used for post-hoc analysis and visualization purposes only, and were not the dependent variable in the primary rmANOVA.

## Results

One participant withdrew during the first session due to concerns over the overall testing duration, while another discontinued participation because of severe vomiting. Data were presented from the remaining 27 participants (age range: 18–66 years; mean ± SD: 34.48 ± 15.75; 18 females).

### Resting motor threshold (RMT)

A two-way repeated-measures ANOVA with factors Drug (baclofen vs. placebo) and Time (PRE vs. POST) was conducted on RMT values. Descriptive statistics showed that RMT was 64.00 ± 5.86 for baclofen PRE, 64.70 ± 5.70 for baclofen POST, 64.22 ± 6.12 for placebo PRE, and 65.19 ± 4.97 for placebo POST. The main effect of Time was significant (F(1,26) = 10.484, *p* = 0.003, partial η^2^ = 0.287), indicating that RMT increased from PRE to POST regardless of drug condition. However, neither the main effect of Drug (F(1,26) = 0.588, *p* = 0.450, partial η^2^ = 0.022) nor the Drug × Time interaction (F(1,26) = 0.321, *p* = 0.576, partial η^2^ = 0.012) was significant. These results suggest that baclofen did not differentially affect RMT compared to placebo, and that the observed ISP effects are unlikely to be confounded by changes in stimulation intensity.

### TMS-induced ISP in the motor region

We calculated change scores for interhemispheric signal propagation (POST-PRE). There was a significant time × group interaction on ISP (*F*_*(1,52)*_ = 4.57, *p* = 0.037, *partial η²* = 0.081) (Fig 2). Post-hoc analysis indicated that baclofen significantly reduced ISP compared to placebo (ΔISP_baclofen_ = −19.60%, ΔISP_placebo_ = 20.19%, *p* = 0.037).

**Fig 2:**
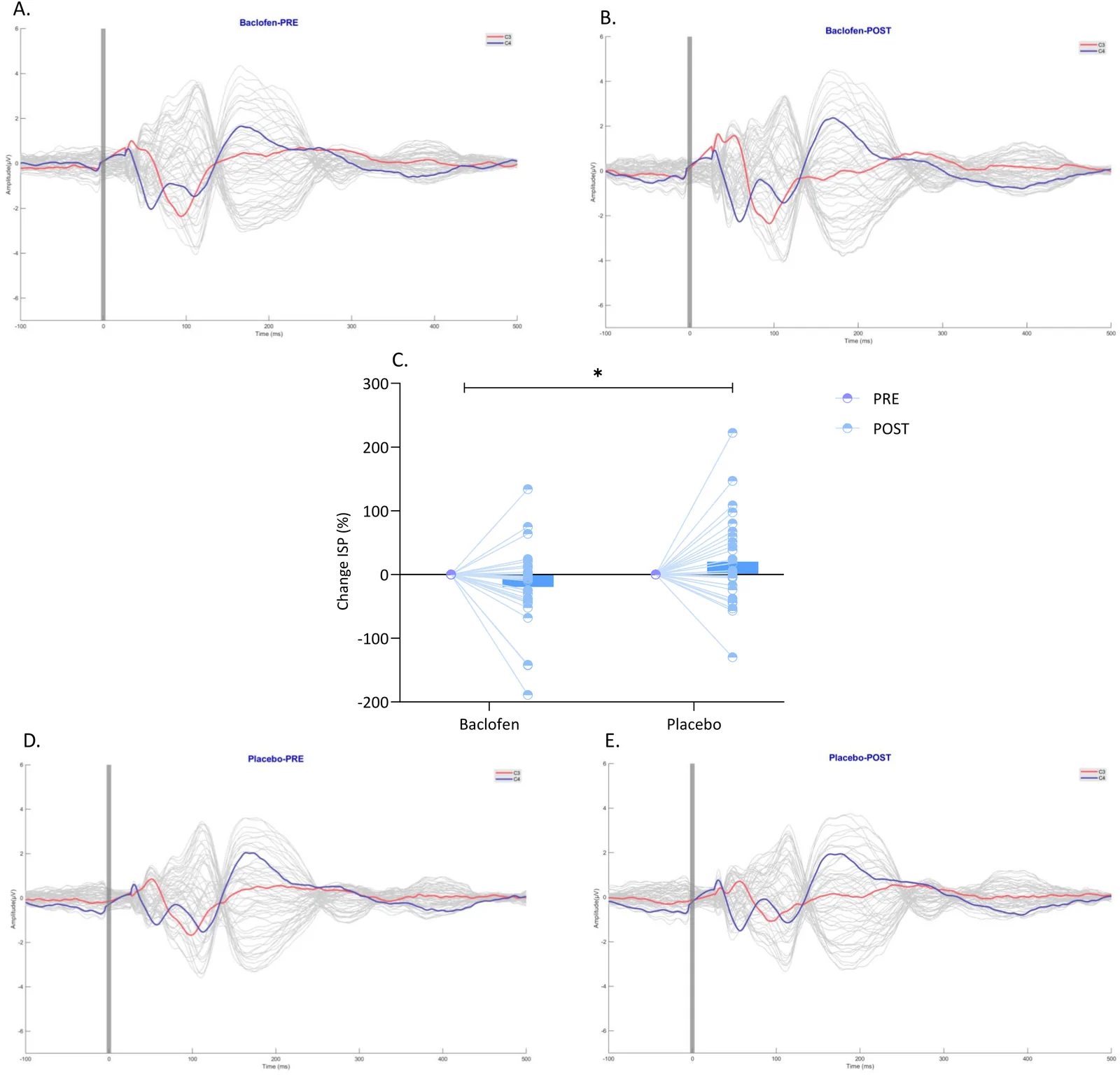
Results in the motor region. Butterfly plots of TEPs recorded before (A) and after (B) oral administration of baclofen, with red and blue lines indicating the left (C3) and right (C4) motor region electrodes, respectively; the gray shading marks the timing of the TMS pulses. C Changes in interhemispheric signal propagation (ISP%) following baclofen or placebo administration, calculated as POST minus PRE. The PRE time point is set to zero as a visual reference for ‘no change from baseline,’ reflecting the mathematical definition of a change score (Δ = POST − PRE). Statistical analysis was performed on raw ISP values using repeated-measures ANOVA (see Methods). Baclofen significantly reduced ISP compared to placebo. D and E display butterfly plots of TEPs before and after placebo administration, using the same conventions for electrode coloring and TMS pulse timing. *p < 0.05.

To control for potential confounding effects of global brain activity and sensory artifacts, we examined Cz butterfly plot and performed an additional control analysis in which the change score (POST − PRE) of Cz was entered as a covariate in the repeated-measures ANOVA. The Cz electrode, located at the midline vertex, is sensitive to global EEG fluctuations and non-specific sensory inputs, and was therefore selected a priori as an index of global cortical activity. The butterfly plot showed that Cz had a clear 100–200 ms complex close to the sensory artefacts (S4 Fig). Statistical analyses indicated that Cz covariate did not significantly predict ISP changes (*F*_*(1,51)*_ = 1.138, *p* = 0.291). Critically, the time × group interaction on ISP remained significant after controlling for Cz activity (*F*_*(1,51)*_ = 4.126, *p* = 0.047, *partial η²* = 0.075), indicating that the baclofen-induced reduction in ISP cannot be fully accounted for by global EEG changes or non-specific sensory influences.

### TMS-induced ISP in the dorsolateral prefrontal cortex

A two-way repeated-measures ANOVA revealed no significant time × group interaction on DLPFC ISP (*F*_*(2,78)*_ = 1.721, *p* = 0.186, *partial η²* = 0.042, Fig 3).

**Fig 3:**
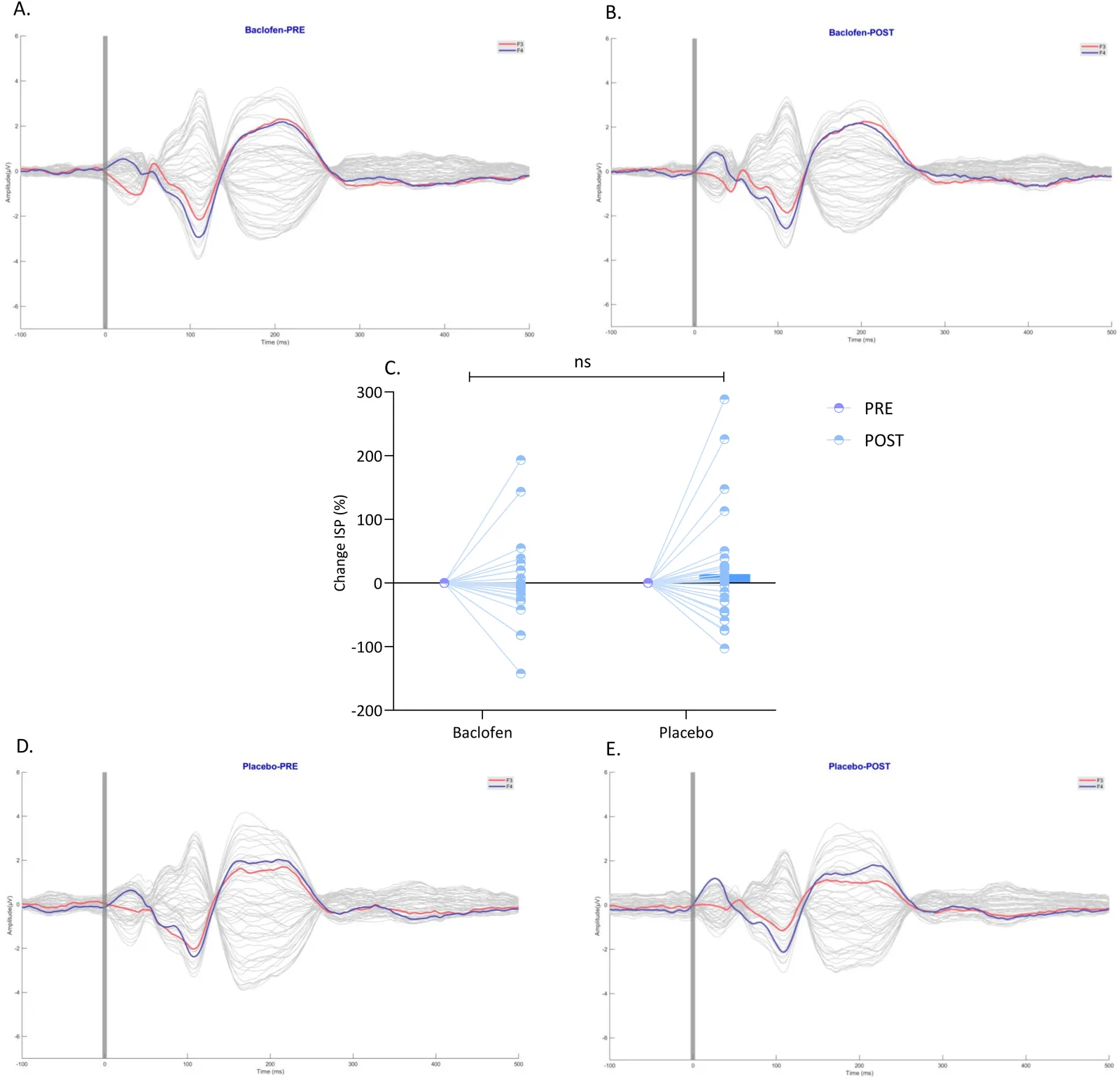
Results in the dorsolateral prefrontal region. A and B depict butterfly plots of TEPs obtained before (A) and after (B) oral administration of baclofen. Red and blue traces represent the left (F3) and right (F4) dorsolateral prefrontal cortex (DLPFC) region electrodes, respectively; the gray shading marks the timing of the TMS pulses. C Changes in interhemispheric signal propagation (ISP%) following baclofen or placebo administration, calculated as POST minus PRE. The PRE time point is set to zero as a visual reference for ‘no change from baseline,’ reflecting the mathematical definition of a change score (Δ = POST − PRE). Statistical analysis was performed on raw ISP values using repeated-measures ANOVA (see Methods). No significant difference was observed between baclofen and placebo. D and E illustrate TEPs recorded before and after placebo administration, following the same electrode and shading conventions. ns = not significant.

## Discussion

By demonstrating that baclofen significantly modulates ISP in a region-specific manner, the present study achieves both aims: it independently replicates the GABA_B_ mechanism of interhemispheric inhibition [13], and validates ISP as a pharmacologically sensitive marker for probing GABA_B_ergic neurotransmission.Given the critical role of interhemispheric communications, our study independently replicated the mechanisms of GABA_B_ receptor neurotransmission. Specifically, baclofen increased interhemispheric inhibition indexed by decreased ISP over the motor region. Moreover, we more than doubled the sample size of the original baclofen-ISP study [13], further yielding the pharmacological mechanisms of interhemispheric inhibition. Understanding the mechanisms of interhemispheric communications is important to uncover the effects of brain stimulation technologies. For instance, lesions to one hemisphere could change interhemispheric communications and lead to dysfunctional motor performances. Contralateral inhibitory rTMS stimulation is suggested to have a positive effect on motor performance [26,37–40]. Our findings of interhemispheric inhibition thus could help explain the effects of rTMS treatment.

It is noted that the effects of baclofen on interhemispheric inhibition could be mediated by both presynaptic and postsynaptic effects at the level of local inhibitory circuits. Baclofen does not selectively act on subunits of the GABA_B_ receptor. There is evidence that callosal inhibition relies on postsynaptic GABA_B_ergic neurotransmission [41]. At the same time, the disinhibitory effect of paired-pulse TMS is consistent with those mechanisms mediated by presynaptic GABA_B_ receptors [32]. Although callosal fibres are mostly excitatory [42], some callosal projection neurons are GABAergic in nature [43]. This was also demonstrated in a previous TMS-EEG study in which baclofen is believed to increase GABA_B_ receptor activity [4]. Therefore, it is possible that these GABAergic neurons were directly activated by baclofen to increase interhemispheric inhibition.

As the ISP does not rely on motor output, it can be applied to a range of cortices beyond the motor region. This advantage largely extends the application of interhemispheric assessment in the pathology of disorders as well as the mechanisms of brain stimulation technologies. Moreover, some previous studies did not establish a modulatory effect of baclofen in motor cortical interhemispheric inhibition(IHI) [4]. Meanwhile, a consistent modulation of ISP was identified in our study and the literature [13]. It is likely that ISP directly probes cortico-cortical connectivity while IHI relies on motor output. These findings together highlight that ISP may be a more sensitive measure of interhemispheric activity.

Our findings have direct implications for clinical practices. Impaired interhemispheric communication has been implicated in the pathophysiology of various chronic conditions [32,36,44], though its precise functional contributions remain unclear. The TMS-EEG derived ISP measure offers a reliable method to investigate these interactions. Clinically, our results suggest that ISP could serve as a biomarker to optimise rTMS treatment strategies. Specifically, ISP measurements may help quantify rTMS-induced contralateral inhibition effects and guide the design of stimulation protocols (e.g., theta-burst stimulation [24] or accelerated iTBS) for specific disorders [45–47], potentially improving therapeutic outcomes.

It is interesting to note that baclofen-induced ISP decrease was not evidenced in the prefrontal region. There is physiological evidence that highly lateralised motor functions require IHI to coordinate motor movements and produce a unified motor output [42]. Meanwhile, there may be a greater balance between inhibition and excitation in the prefrontal region [see discussions in 13]. In addition, callosal communication between the prefrontal cortices involves complex interactions between glutamate, GABA, and dopamine-sensitive mechanisms [10], indicating the complexity of interhemispheric prefrontal projections. Several methodological limitations may explain the null finding in the DLPFC. First, Beam F3 localization lacks MRI-guided precision, and individual anatomical variability may have resulted in suboptimal targeting. Second, 110% RMT intensity may be suboptimal for DLPFC given its greater scalp-to- region distance and different optimal coil orientation. These factors may reduced sensitivity to detect prefrontal drug effects.

There were some limitations in this study. Regarding the choice of baclofen dose, we acknowledge that previous work [13] used a higher dose of 50 mg in their original TMS-EEG study. However, the 10 mg dose was selected in the present study for several reasons. First, 10 mg is the standard oral dose of baclofen used in human pharmacological studies in healthy volunteers, balancing safety, tolerability, and pharmacodynamic sensitivity. Second, previous TMS-EEG work has successfully demonstrated GABA_B_-mediated effects on TMS-evoked EEG potentials using the same 10 mg dose [21,22]. Third, the primary goal of the present study was to validate ISP as a sensitive pharmacodynamic marker in a larger sample, which required a dose with established neurophysiological effects rather than the maximal tolerable dose. Consistent with established protocols [21,22], we implemented a 90-minute waiting period prior to post-drug TMS-EEG recordings. This duration was selected to ensure that TMS-EEG assessments coincided with peak or near-peak plasma baclofen concentrations, which typically occur 0.5–1.5 hours following oral administration, and to maintain consistency with the previous work [13] that this study aims to replicate. Plasma concentration peaks were not systematically monitored due to the absence of blood sampling. Notably, our observed TEP amplitudes remained below ±4 μV, substantially lower than the typical artifact thresholds (>±10 μV) reported for late TEP components (>100 ms) in central electrode regions [48,49]. To enhance signal specificity, ISP was quantified as a right-to-left hemisphere activation ratio, a method shown to effectively minimise artifact contributions [7]. While our experimental setup did not incorporate a neuronavigation system for precise DLPFC targeting, we employed the validated Beam F3 method [25], which provides reliable targeting accuracy comparable to navigation-assisted approaches. In terms of stimulation intensity, we adjusted the resting motor threshold (RMT) following drug administration—a protocol that remains debated in the field [22]. Additionally, while our interpolation window of −5–25 ms is longer than the minimal amplifier recovery time of 5–12 ms reported in some studies [50], this conservative approach was adopted to ensure complete removal of secondary muscle artifacts that are particularly prominent when stimulating lateral cortical regions such as M1 and DLPFC [51,52]. Previous work [51] demonstrated that stimulation over lateral scalp positions produces a large secondary artifact that only fully recovers after approximately 25–40 ms, which is consistent with our interpolation window. Importantly, as our ISP measure focuses on the 50–160 ms post-stimulus window [7,13], this interpolation does not compromise the neural signals central to our research question.Importantly, previous research has demonstrated good consistency between RMT measurements obtained with and without this adjustment [22], supporting the validity of our approach. An additional limitation concerns potential sensory confounds in TMS-EEG recordings. TMS stimulation produces auditory and somatosensory inputs that can evoke peripheral responses contaminating cortical signals [53]. Although we employed noise masking, this method does not eliminate auditory evoked potentials. We did not use additional control methods such as foam padding between the coil and scalp to dampen somatosensory responses. Consequently, the observed TEPs may reflect a mixture of direct cortical activation and sensory-evoked activity. However, the ratio-based ISP measure and the specificity of drug effects to the motor region (with null effects in DLPFC) argue against a purely sensory explanation. S5 Fig illustrates the spatiotemporal evolution of voltage distribution over time, revealing that early components (P30, N45) exhibit localized distributions over the central-parietal region, in contrast to the fronto-central diffuse topography of N100-P180. This spatiotemporal differentiation suggests that these components may originate from distinct neural sources. While the fronto-central distribution of N100-P180 overlaps with auditory evoked potential topographies, the more localized, condition-specific patterns of early components support the interpretation that the ISP measure captures at least partially TMS-specific cortical activation rather than purely sensory contamination. We acknowledge that the use of suprathreshold stimulation intensity (110% RMT) over M1 means that sensory reafference from motor evoked potentials may have confounded the TEP. It is noted that our ISP measure is calculated as a ratio of contralateral to ipsilateral hemisphere activation, which may reduce bilateral sensory contributions, as sensory reafference would affect both hemispheres. However, this indirect consideration does not substitute for direct experimental controls. Future studies should incorporate more sophisticated sensory deprivation methods to further disentangle the contributions of direct cortical activation and sensory-evoked activity. To further investigate the effects of baclofen on local cortical excitability, we performed cluster-based permutation tests on TEP waveforms. In the M1 region, no significant differences were found between the baclofen and placebo groups across all predefined time windows (P25, N45, P60, N100, P180; all *p* > 0.05). In the DLPFC region, a significant positive cluster was detected only in the P60 time window (*p* = 0.036), indicating that TEP amplitudes in the baclofen group were significantly higher than those in the placebo group during this time window. No significant group differences were observed in the DLPFC for the other time windows (P25, N45, N100, P180; all *p* > 0.05). These results suggest that the effects of baclofen on local cortical excitability may be region- and time-specific. However, within-group comparisons revealed no significant pre-post changes in the baclofen group for either the M1 or DLPFC regions across all predefined time windows (P25, N45, P60, N100, P180; all *p* > 0.05, cluster-corrected). This suggests that the observed group difference in the DLPFC P60 component may reflect baseline discrepancies between groups rather than a genuine drug effect. Alternatively, it could be driven by changes in the placebo group, such as practice effects or expectation effects. In addition, the number of components removed in the first ICA round reflects the cumulative total of artifactual components (~9–14 components). There is a possibility that it may have been overly aggressive for a first round of ICA.

## Conclusion

This study demonstrated the pharmacological mechanisms of interhemispheric inhibition as indexed by ISP. This finding highlights the potential of ISP for characterising interhemispheric communication, which could inform the treatment for a series of disorders with dysfunctional callosal transmission.

## Supporting information

S1 Fig The figure shows the averaged TMS-EEG signal (electrode Fz) from a representative participant under DLPFC stimulation. (TIFF)

S2 Fig Representative example of manual component inspection during the first ICA round. The figure shows a comparison of artifactual components identified for removal and neural components retained during the first ICA round for a representative participant, including component waveforms, scalp topographies, and power spectra. The removed components exhibit characteristic features of TMS-related artifacts (e.g., sharp waveforms, focal scalp distributions, and spectral profiles consistent with known artifact patterns), whereas the retained components preserve typical TEP waveform features. Similar manual visual inspections were performed for all participants and all conditions.
(TIFF)

S3 Fig Representative example of EEG signal before and after preprocessing. The figure illustrates the effectiveness of the artifact removal procedure across all conditions, showing averaged TEPs before preprocessing (upper panel) and after preprocessing (lower panel) from a representative participant.
(TIF)

S4 Fig Butterfly plots of TEPs at the Cz electrode before and after drug administration. (A) Pre-baclofen and (B) post-baclofen TEP butterfly plots; (C) pre-placebo and (D) post-placebo TEP butterfly plots. The Cz electrode is highlighted in purple, and the vertical gray bar indicates the timing of the TMS pulse.
(TIFF)

S5 Fig Topographical voltage distribution for the peaks of interest. The figure illustrates the spatiotemporal evolution of voltage distribution over time, with early components (P30, N45) showing localized central-parietal distributions and late components (N100, P180) showing fronto-central diffuse topography.
(TIF)
