# Supplementary material for: Concurrent TMS-EEG to characterise interhemispheric signal propagation over the motor and prefrontal regions: An independent replication of GABAB receptor mechanisms
Source: PLoS One. 2026 Oct 16;21(10):e0360250. doi: 10.1371/journal.pone.0360250 (PMC13674705; doi:10.1371/journal.pone.0360250)
Supplement: Supplementary file 2 [file pone.0360250.s008.docx]

**Dear Editor and Reviewers,**

We sincerely thank the Editor and the reviewers for their thoughtful and constructive comments on our manuscript (PONE-D-25-38064). These suggestions have greatly helped us improve the quality of our work. Our revisions are as follows.

**Reviewer #1: I greatly appreciate the authors’ efforts in revising the manuscript. The paper has improved substantially, and the authors have addressed all of the concerns raised in my previous review in a satisfactory manner.**

**I have no further major comments. I only have a few minor suggestions:**

**1) I suggest adapting the scale in Fig. S1 so that the EEG response to TMS (albeit largely artifactual) is visible and can be directly compared with the preprocessed response. As a reference, the authors may wish to consult Fig. S3 in the already cited paper by Zazio et al. (2022).**

**2) I suggest removing “consistent with established TMS-EEG protocols” (line 127), as well as the acronym iSP for ipsilateral silent period (line 51), as these may be misleading.**

Response: We thank the reviewer for the positive feedback and for these helpful suggestions. We have carefully addressed both points as follows:

Regarding Fig. S1 (now Fig. S3): We have adapted the scale of the figure to ensure that the raw EEG response to TMS is clearly visible and can be directly compared with the preprocessed response, following the reviewer's suggestion and the format of Fig. S3 in Zazio et al. (2022).

**Fig. S3 Representative example of EEG signal before and after preprocessing.**

The figure illustrates the effectiveness of the artifact removal procedure

across all conditions, showing averaged TEPs before preprocessing (upper panel)

and after preprocessing (lower panel) from a representative participant.

Regarding the suggested deletions: We have removed the phrase "consistent with established TMS-EEG protocols" from line 127(now line 119) in the Methods section. We have also removed the acronym "iSP" for ipsilateral silent period from line 51(now line 45) in the Introduction.

These changes have been made in the revised manuscript. We hope these revisions meet the reviewer's expectations

**Reviewer #2: The authors have now adequately addressed my specific comments. I have no further questions for the authors.**

Response: We sincerely thank the reviewer for the positive acknowledgment and for their valuable time and effort in reviewing our work.

**Reviewer #3**

**1. In response to my first comment, the authors refer to recent review that shows a suprathreshold intensity over M1 is common, and by extension acceptable. However, it’s prevalence in existing literature is not evidence that reflects good practice. Indeed, the review cited by the authors goes on to describe in detail why it is problematic (points that support my previous comment). As far as I can tell, the authors have in no way addressed this concern.**

Response: We agree with you that the available data cannot address this concern. We have now acknowledged this limitation.

“We acknowledge that the use of suprathreshold stimulation intensity (110% RMT) over M1 means that sensory reafference from motor evoked potentials may have confounded the TEP. It is noted that our ISP measure is calculated as a ratio of contralateral to ipsilateral hemisphere activation, which may reduce bilateral sensory contributions, as sensory reafference would affect both hemispheres. However, this indirect consideration does not substitute for direct experimental controls. Future studies should incorporate more sophisticated sensory deprivation methods to further disentangle the contributions of direct cortical activation and sensory-evoked activity.” Page 17, Line 390-397

**2. The authors state that ‘a sufficient number of trials’ were included in their TEP analysis, but do not provide any information on how they justify what they view as sufficient.**

Response: Thank you for this critical methodological point. To justify the “sufficient number of trials” claim, we now provide literature-based evidence in the revised Methods section.

“To justify the sufficient number of trials, established TMS-EEG literature was referred. For the M1, Lioumis et al. (2009) reported a high reproducibility (r > 0.80) with ~100 pulses per condition and at least 80 epochs retained for averaging [35]. For the DLPFC, Noda et al. (2024) demonstrated that 80 pulses yield TEP waveforms equivalent to 160 or 240 pulses, while 40 pulses are insufficient [36]. In our dataset, the retained trial numbers are sufficient for reliable TEP estimation.” Page 9, Line 199- Page 10, Line 205

**3. In response to my fourth comment, the authors refer to older literature to suggest that the stimulus artifact may last as long as 40ms. In the current state of the art, this should not be the case unless there is a problem with the recording. Despite this, the most effective approach for identifying the temporal window to interpolate would be to examine the length of the artifact in their own data.**

Response: We thank the reviewer for this important methodological comment. We have now examined the artifact duration in our own data.

“The -5 to 25 ms interpolation window was selected based on direct inspection of the TMS-related artifact in this dataset. While the initial amplifier recovery occurred within approximately 5–12 ms, a secondary artifact component arising from cranial muscle activation was observed to persist up to approximately 20–25 ms, with considerable inter-individual variability in its duration. To ensure complete and consistent removal of the full artifact time course for all participants, a conservative fixed window of -5 to 25 ms was applied. A representative example of the artifact and the interpolation window is provided in Fig. S1.” Page 8, Line 162-169

**Fig. S1** The figure shows the averaged TMS-EEG signal (electrode Fz) from a representative participant under DLPFC stimulation.

**4. The responses to my sixth and seventh comments only serve to further suggest that the data contributing to this paper are likely to be highly reflective of contamination from sensory artifacts.**

Response: We thank the reviewer for this critical observation. We fully acknowledge that sensory confounds represent a major challenge in TMS-EEG research. In our revised manuscript, we have now taken additional steps to empirically address this concern. We have now examined the influence of Cz, which is sensitive to global EEG fluctuations and non-specific sensory inputs, on our main findings.

“To control for potential confounding effects of global brain activity and sensory artifacts, we examined Cz butterfly plot and performed an additional control analysis in which the change score (POST − PRE) of Cz was entered as a covariate in the repeated-measures ANOVA. The Cz electrode, located at the midline vertex, is sensitive to global EEG fluctuations and non-specific sensory inputs, and was therefore selected a priori as an index of global cortical activity. The butterfly plot showed that Cz had a clear 100-200 ms complex close to the sensory artefacts (Fig. S4). Statistical analyses indicated that Cz covariate did not significantly predict ISP changes (*F_(1,51)_* = 1.138, *p* = 0.291). Critically, the time × group interaction on ISP remained significant after controlling for Cz activity (*F_(1,51)_* = 4.126, *p* = 0.047, *partial η²* = 0.075), indicating that the baclofen-induced reduction in ISP cannot be fully accounted for by global EEG changes or non-specific sensory influences.” Page 12, Line 261-271

**Fig. S4 Butterfly plots of TEPs at the Cz electrode before and after drug administration.** (A) Pre-baclofen and (B) post-baclofen TEP butterfly plots; (C) pre-placebo and (D) post-placebo TEP butterfly plots. The Cz electrode is highlighted in purple, and the vertical gray bar indicates the timing of the TMS pulse.

Response: We have also acknowledged the importance of sensory deprivation methods in future studies.

“Future studies should incorporate more sophisticated sensory deprivation methods to further disentangle the contributions of direct cortical activation and sensory-evoked activity.” Page 17, Line 395-397

**5. Regarding the ICA process, it seems that a large number of components were removed, which would increase the possibility the neural activity of interest was unintentionally removed. In addition, the first ICA round removed on average between ~9 and 14 components. This round is only intended to remove large artifacts associated with the tail of TMS artifact, which generally is achieved by removing 1-2 components. Taken together, it seems that the ICA implementation may have been overly aggressive.**

Response: We thank the reviewer for raising this important methodological concern. In our pipeline, we manually inspected every single component marked for removal at each ICA round to ensure that only artifactual components were removed while neural signals were preserved. To illustrate this, we have now included a supplementary figure (Fig. S2) in the revised manuscript, showing an example of the manual inspection performed during the first ICA round. As shown in the figure, the components identified for removal exhibited characteristic features of TMS-related artifacts (e.g., sharp waveforms, focal scalp topographies, and spectral profiles consistent with known artifact patterns). This manual inspection procedure was applied to all participants and all conditions, ensuring the accuracy of artifact rejection while minimizing the risk of inadvertently removing neural activity. We have now added the following clarification in the Methods section.

“The number of components removed in the first ICA round reflects the cumulative total of artifactual components identified across multiple ICA runs, with each step manually inspected to confirm that only artifactual components were removed while neural signals were retained (Fig. S2).” Page 8, Line 178- Page 9, Line 181

**Fig. S2 Representative example of manual component inspection during the first ICA round.** The figure shows a comparison of artifactual components identified for removal and neural components retained during the first ICA round for a representative participant, including component waveforms, scalp topographies, and power spectra. The removed components exhibit characteristic features of TMS-related artifacts (e.g., sharp waveforms, focal scalp distributions, and spectral profiles consistent with known artifact patterns), whereas the retained components preserve typical TEP waveform features. Similar manual visual inspections were performed for all participants and all conditions.

Response: We have also discussed the possibility of an overly aggressive first round of ICA.

“In addition, the number of components removed in the first ICA round reflects the cumulative total of artifactual components (~9-14 components). There is a possibility that it may have been overly aggressive for a first round of ICA.” Page 18, Line 412-414

Overall, we really appreciate the reviewers for providing these great suggestions. This work is now much improved. We wish these changes are satisfactory.
