# Supplementary material for: Concurrent TMS-EEG to characterise interhemispheric signal propagation over the motor and prefrontal regions: An independent replication of GABAB receptor mechanisms
Source: PLoS One. 2026 Oct 16;21(10):e0360250. doi: 10.1371/journal.pone.0360250 (PMC13674705; doi:10.1371/journal.pone.0360250)
Supplement: Peer review history

# Peer review: Decision Letter 0

**DOI:** 10.1371/journal.pone.0360250.r001

**Authors:** Fengxue Qi

13 Feb 2026

Dear Dr. Che,

plosone@plos.org

- A letter that responds to each point raised by the academic editor and reviewer(s). You should upload this letter as a separate file labeled 'Response to Reviewers'.
- A marked-up copy of your manuscript that highlights changes made to the original version. You should upload this as a separate file labeled 'Revised Manuscript with Track Changes'.
- An unmarked version of your revised paper without tracked changes. You should upload this as a separate file labeled 'Manuscript'.

If you would like to make changes to your financial disclosure, please include your updated statement in your cover letter. Guidelines for resubmitting your figure files are available below the reviewer comments at the end of this letter.

If applicable, we recommend that you deposit your laboratory protocols in protocols.io to enhance the reproducibility of your results. Protocols.io assigns your protocol its own identifier (DOI) so that it can be cited independently in the future. For instructions see: https://journals.plos.org/plosone/s/submission-guidelines#loc-laboratory-protocols. Additionally, PLOS ONE offers an option for publishing peer-reviewed Lab Protocol articles, which describe protocols hosted on protocols.io. Read more information on sharing protocols at https://plos.org/protocols?utm_medium=editorial-email&utm_source=authorletters&utm_campaign=protocols.

We look forward to receiving your revised manuscript.

Kind regards,

Fengxue Qi, Ph.D.

Academic Editor

PLOS One

Journal Requirements:

When submitting your revision, we need you to address these additional requirements.

1.Please ensure that your manuscript meets PLOS ONE's style requirements, including those for file naming. The PLOS ONE style templates can be found at

https://journals.plos.org/plosone/s/file?id=wjVg/PLOSOne_formatting_sample_main_body.pdf and

https://journals.plos.org/plosone/s/file?id=ba62/PLOSOne_formatting_sample_title_authors_affiliations.pdf

2. We noticed you have some minor occurrence of overlapping text with the following previous publication(s), which needs to be addressed:

https://www.nature.com/articles/s41386-019-0468-7

In your revision ensure you cite all your sources (including your own works), and quote or rephrase any duplicated text outside the methods section. Further consideration is dependent on these concerns being addressed.

3. Thank you for stating the following financial disclosure:

“XC was supported by the Zhejiang Provincial Natural Science Foundation of China (LY23C090002), and the Open Research Fund of the Zhejiang Key Laboratory of Precision Psychiatry (2025A2).”

Please state what role the funders took in the study.  If the funders had no role, please state: "The funders had no role in study design, data collection and analysis, decision to publish, or preparation of the manuscript."

If this statement is not correct you must amend it as needed.

Please include this amended Role of Funder statement in your cover letter; we will change the online submission form on your behalf.

4. In the online submission form you indicate that your data is not available for proprietary reasons and have provided a contact point for accessing this data. Please note that your current contact point is a co-author on this manuscript. According to our Data Policy, the contact point must not be an author on the manuscript and must be an institutional contact, ideally not an individual. Please revise your data statement to a non-author institutional point of contact, such as a data access or ethics committee, and send this to us via return email. Please also include contact information for the third party organization, and please include the full citation of where the data can be found.

5. We notice that your supplementary figures are uploaded with the file type 'Figure'. Please amend the file type to 'Supporting Information'. Please ensure that each Supporting Information file has a legend listed in the manuscript after the references list.

6. If the reviewer comments include a recommendation to cite specific previously published works, please review and evaluate these publications to determine whether they are relevant and should be cited. There is no requirement to cite these works unless the editor has indicated otherwise.

Additional Editor Comments:

[Note: HTML markup is below. Please do not edit.]

Reviewer's Responses to Questions

**Comments to the Author**

1. Is the manuscript technically sound, and do the data support the conclusions?

Reviewer #1: Partly

Reviewer #2: Yes

Reviewer #3: No

**********

2. Has the statistical analysis been performed appropriately and rigorously?

Reviewer #1: Yes

Reviewer #2: Yes

Reviewer #3: No

**********

3. Have the authors made all data underlying the findings in their manuscript fully available?

The PLOS Data policy

Reviewer #1: No

Reviewer #2: No

Reviewer #3: No

**********

4. Is the manuscript presented in an intelligible fashion and written in standard English?

Reviewer #1: Yes

Reviewer #2: Yes

Reviewer #3: Yes

**********

Reviewer #1: The present study aims at replicating previous findings on the effects of baclofen on a TMS-EEG-derived measure thought to reflect inter-hemispheric signal propagation (i.e., ISP), obtained both from the primary motor cortex and from the dorso-lateral prefrontal cortex. The results suggest that baclofen reduces the iSP when the motor cortex was stimulated, but not after dorso-lateral prefrontal cortex stimulation.

The study addresses an interesting topic and replication studies are highly valuable to validate innovative measures such as the ones derived from TMS-EEG. However, the work suffers from potential circularity in its rationale and from methodological shortcomings, which limit the strength of the conclusions. These concerns are outlined in detail below.

1) It is unclear whether the study is primarily validating the ISP measure via pharmacological modulation or testing baclofen’s effect on interhemispheric inhibition, introducing interpretative circularity. Such ambiguity is present throughout the paper (abstract, intro, discussion). This is a crucial point that the authors are recommended to address.

2) In the abstract it is stated that the effect “is more prominent over the motor cortex than the prefrontal cortex”. This statement is misleading as the results show non-significant effects when stimulating the prefrontal cortex. I recommend rewording this statement to avoid suggesting a graded effect where only a null finding is present.

3) The acronym ISP standing for interhemispheric signal propagation, although previously used and introduced, is a bit unfortunate in the field, as it may also indicate “ipsilateral silent period”, which is also a measure of transcallosal communication. I recommend to specify this potential ambiguity from the beginning. Moreover, please note that no definition for the acronym IHI has been provided.

4) The replication aim should be emphasized also in the title and/or in the abstract.

5) Methods:

a. Details on EMG recording are missing, which seems to be used at least for rMT calculation. Related to this, I am also wondering whether EMG was recorded also during TMS-EEG sessions, especially during motor cortex stimulation.

b. Please specify whether the order was counterbalanced between experimental conditions.

c. The procedure for stimulation site localization should be described in greater detail, both for M1 and DLPFC. Further details should be provided also for noise masking procedure, as well as for coil orientation (please describe the direction of the current in the brain, e.g. anterior-to-posterior/posterior-to-anterior).

6) Analysis:

a. With a sampling rate of 5 kHz, the TMS artifact typically last about 5 ms (Veniero et al. 2009). Here, the authors reported an interpolation of 25 ms after the TMS pulse. Please provide the rationale of applying such a long time interpolation interval.

b. Please provide additional details on: how many components (range and average) were discarded in every steps; channel interpolation (type and how many channels were interpolated).

c. Related to the previous points, a representative figure showing the averaged signal before and after the preprocessing would be highly informative (even in supplementary materials).

d. It seems inaccurate to refer to the “cortex” when describing the ISP, considering that the analysis is at electrode-level without performing source reconstruction.

7) Results and interpretation

a. Was there any difference in rMT between conditions?

b. Among the limitations of the non-significant finding on DLPFC, a few methodological aspects may be mentioned, such as site localization and TMS intensity (due to differences between M1 in the cortical excitability of this area, scalp-to-cortex distances, optimal coil orientation, etc.)

c. Other TMS measures have been described in the literature as index of transcallosal inhibition, such the ipsilateral silent period (e.g., Meyer et al., 1995) and measures derived from dual coil paradigms (e.g., Ferbert et al., 1992), as well as TMS-EEG measures, such as the M1-P15 TMS-evoked potential component (e.g., Bortoletto et al., 2021; Zazio et al., 2022). This literature should be incorporated in the introduction and/or discussion.

References

- Bortoletto M, Bonzano L, Zazio A, Ferrari C, Pedullà L, Gasparotti R, Miniussi C, Bove M. Asymmetric transcallosal conduction delay leads to finer bimanual coordination. Brain Stimul. 2021 Mar-Apr;14(2):379-388. doi: 10.1016/j.brs.2021.02.002. Epub 2021 Feb 10.

- Ferbert A, Priorit A, Rothwell JC, Day BL, Colebatchi JG, Marsden CD. Interhemispheric inhibition of the human motor cortex. J Physiol 1992.

- Meyer BU, Roricht S, Von Einsiedel HG, Kruggel F, Weindl A. Inhibitory and excitatory interhemispheric transfers between motor cortical areas in normal humans and patients with abnormalities of the corpus callosum. Brain

1995;118:429e40

- Veniero D, Bortoletto M, Miniussi C. TMS-EEG co-registration: on TMS-induced artifact. Clin Neurophysiol. 2009 Jul;120(7):1392-9. doi: 10.1016/j.clinph.2009.04.023. Epub 2009 Jun 16.

- Zazio A, Barchiesi G, Ferrari C, Marcantoni E, Bortoletto M. M1-P15 as a cortical marker for transcallosal inhibition: A preregistered TMS-EEG study. Front Hum Neurosci. 2022 Sep 16;16:937515. doi: 10.3389/fnhum.2022.937515.

Reviewer #2: In this study, the authors using combined transcranial magnetic stimulation and electroencephalography (TMS-EEG) to probe alterations in interhemispheric signal propagation (ISP) across both the motor and prefrontal cortices following oral baclofen administration. Using a double-blind, placebo-controlled, crossover design, they report enhanced inhibition (reduced ISP) following baclofen, largely consistent with its known effects on GABAergic inhibition. This important study extends prior work assessing drug effects (including baclofen) on ISP recorded across motor and prefrontal regions using TMS-EEG; however, importantly it does so in a considerably larger sample. This research is important for understanding the neurochemical mechanisms involved, and helping to validate TMS-EEG as a method to explore these. Overall, this manuscript was clear, concise, and informative, and the methodology and analyses appear sound. I have some specific questions below that I hope the authors will be able to address.

1) I think a little more information on the chosen dose of baclofen (10 mg) would be beneficial, as it is not immediately clear why this does was selected. It is also different to the dose used in the prior Hui et al. 2019 study (50 mg).

2) Can the authors please clarify how randomisation of session order (active, placebo) was determined? Similarly, I think some further justification on the use of the 90-minute waiting period after drug administration is needed – as no bloodwork was performed, it is important to be sure the analysis window captured peak (or close to peak) concentrations of the drug.

3) The authors mention some important limitations of the study, including the lack of MRI-guided neuronaviation for TMS-EEG and lack of blood sample collection. However, another important limitation is in regards to the potential for sensory components of the TMS stimulation (e.g., auditory and somatosensory) to lead to peripherally evoked potentials on the TMS-EEG record (e.g., see: Poorganji et al. [2023]. Scientific Reports, https://www.nature.com/articles/s41598-023-29920-2). While the authors did use noise masking, this does not always completely block out the sound of the TMS coil click. They do not mention using other methods such as foam between the coil and the scalp (to help dampen these responses), so I presume these were not performed? I think this should perhaps be noted as a limitation.

4) I think it would be helpful if the authors were a little more clear in the abstract regarding the results. Specifically, they state “Our findings indicated that baclofen increased interhemispheric inhibition, indexed by decreased ISP. Moreover, this effect was more prominent over the motor cortex than the prefrontal cortex.” This could be interpreted as if there was an effect of baclofen on ISP in the prefrontal cortex, however, the ANOVA was not significant.

Minor

Page 6, line 90: I believe ‘EEP cap’ should be ‘EEG cap’.

Reviewer #3: The study by Zhou and colleagues examined how modulation of GABAB receptor activity influenced the response to TMS-evoked EEG potentials (TEP) generated by stimulation to dorsolateral prefrontal cortex (DLPFC) and primary motor cortex (M1). This was achieved by recording TEPs before and after oral intake of baclofen or placebo. The authors report a drug-related change in an index of interhemispheric communication. This paper is a replication of an older study, implemented with an increased sample size. While conceptually interesting, several elements of the methodology are not consistent with current best practice, which raises concerns about outcomes. In addition, several elements of the methods are unclear. Specific comments are listed below.

1. Stimulation appears to have been applied at 110% RMT for both sites. While this is fine for DLPFC, the suprathreshold intensity over M1 means that sensory reafference from motor evoked potentials would have confounded the TEP.

2. A total of 105 trials were collected, which would presumably mean that a lower number were included in the analysis. This is low for current standards. Descriptives of final trial numbers should be reported.

3. Apologies if I missed it, but please specify the EEG amplifier that was used.

4. The TMS-related artifact peak was removed by excising data from -5 to 25 ms, relative to TMS application. The width of this window would have resulted in removal of neural activity associated with local cortical activation (i.e.,P25/30).

5. The approach to the analysis is unclear. The methods section initially states that raw ISP values were compared between conditions and time points using rmANOVA. However, the results section then refers to calculation of change values that then appear to be the focus of the results. This needs to be clarified. In addition, details of how the change values were calculated, and how they were analysed statistically, should be provided. The plots in Fig 2C/3C appear to indicate that the analysis of change data included both Pre and Post timepoints (i.e., the Pre point is just 0), which isn’t appropriate.

6. The derivation of ISP values focusses on TEP data in the 50-150ms time window. This period is widely recognised within the current literature as likely reflecting indirect activity generated by sensory input (particularly auditory potentials). Consequently, it is highly likely that the reported results are confounded to some extent (possibly extensively) by sensory input.

7. Examination of butterfly plots shows that all conditions appear to contain responses that typify sensory contamination. Specifically, the signals are dominated by large N100-P180 complexes and have small, poorly differentiated early peaks. Examination of component topographies would help elucidate the source of these components, with fronto-central distributions providing further indication for the presence of sensory contamination. Given this, and without any control conditions, it remains difficult to identify the extent to which the reported data reflect direct vs indirect (sensory) brain responses.

8. Between-group comparisons of baseline TEPs do not appear to be provided. These should be reported to demonstrate data are not confounded by baseline differences in responses. Comparisons of other baseline characteristics should also be reported, including RMT and descriptives of analysis (i.e., number of epochs and ICs excluded).

9. It would be of interest to also investigate global changes in the TEP due to drug. Standard approaches would involve cluster-based comparisons targeting the early TEP potentials.

**********

what does this mean?). If published, this will include your full peer review and any attached files.

If you choose “no”, your identity will remain anonymous but your review may still be made public.

**Do you want your identity to be public for this peer review?**  For information about this choice, including consent withdrawal, please see our Privacy Policy

Reviewer #1: No

Reviewer #2: No

Reviewer #3: No

**********

[NOTE: If reviewer comments were submitted as an attachment file, they will be attached to this email and accessible via the submission site. Please log into your account, locate the manuscript record, and check for the action link "View Attachments". If this link does not appear, there are no attachment files.]

To ensure your figures meet our technical requirements, please review our figure guidelines: https://journals.plos.org/plosone/s/figures

You may also use PLOS’s free figure tool, NAAS, to help you prepare publication quality figures: https://journals.plos.org/plosone/s/figures#loc-tools-for-figure-preparation.

NAAS will assess whether your figures meet our technical requirements by comparing each figure against our figure specifications.

# Author comment: Author response to Decision Letter 1

**DOI:** 10.1371/journal.pone.0360250.r002

19 Apr 2026

Dear Editor and Reviewers,

We sincerely thank the Editor and the reviewers for their thoughtful and constructive comments on our manuscript (PONE-D-25-38064). These suggestions have greatly helped us improve the quality of our work. Our revisions are as follows.

Reviewer #1: The present study aims at replicating previous findings on the effects of baclofen on a TMS-EEG-derived measure thought to reflect inter-hemispheric signal propagation (i.e., ISP), obtained both from the primary motor cortex and from the dorso-lateral prefrontal cortex. The results suggest that baclofen reduces the iSP when the motor cortex was stimulated, but not after dorso-lateral prefrontal cortex stimulation.

The study addresses an interesting topic and replication studies are highly valuable to validate innovative measures such as the ones derived from TMS-EEG. However, the work suffers from potential circularity in its rationale and from methodological shortcomings, which limit the strength of the conclusions. These concerns are outlined in detail below.

1) It is unclear whether the study is primarily validating the ISP measure via pharmacological modulation or testing baclofen’s effect on interhemispheric inhibition, introducing interpretative circularity. Such ambiguity is present throughout the paper (abstract, intro, discussion). This is a crucial point that the authors are recommended to address.

Response: We thank the reviewer for raising this important point regarding potential circularity in the study's rationale. We acknowledge that the original manuscript did not clearly distinguish between the two interrelated but distinct aims. This ambiguity has been carefully addressed in the revised manuscript. Specifically, we have made the following changes:

We have revised the sentence the Abstract: " This study aimed to validate the sensitivity of a TMS-EEG-based measure of interhemispheric signal propagation (ISP) to GABAB receptor modulation, while independently replicating the pharmacological mechanisms of interhemispheric inhibition." This clarifies that ISP validation and pharmacological replication are complementary, rather than circular, objectives. Page 2, line 19-22

We have explicitly listed the two complementary aims in the Introduction: " The current study was designed with two complementary aims: (1) to conduct an independent replication of GABAB receptor effects on ISP with an expanded sample size; and (2) to validate ISP as a sensitive pharmacodynamic marker for GABAergic modulation across cortical regions. Page 4, line 68-73

We have added a clear statement that the study achieves both aims in the Discussion: “By demonstrating that baclofen significantly modulates ISP in a region-specific manner, the present study achieves both aims: it independently replicates the GABAB mechanism of interhemispheric inhibition[13], and validates ISP as a pharmacologically sensitive marker for probing GABABergic neurotransmission.” Page 12, line 271-274

2) In the abstract it is stated that the effect “is more prominent over the motor cortex than the prefrontal cortex”. This statement is misleading as the results show non-significant effects when stimulating the prefrontal cortex. I recommend rewording this statement to avoid suggesting a graded effect where only a null finding is present.

Response: We thank the reviewer for this important suggestion. We agree that the original phrasing was not appropriate, as it could imply a graded or comparative effect where in fact the prefrontal cortex results were non-significant. We have therefore revised the sentences to accurately reflect the statistical findings.

The original sentence has been replaced with the following: “Our findings indicated that baclofen increased interhemispheric inhibition over the motor region, indexed by significantly decreased ISP (p = 0.037). No significant effect was observed in the dorsolateral prefrontal region (p = 0.186), suggesting regional specificity in GABAB receptor modulation of ISP.” Page 2, line 24-27

3) The acronym ISP standing for interhemispheric signal propagation, although previously used and introduced, is a bit unfortunate in the field, as it may also indicate “ipsilateral silent period”, which is also a measure of transcallosal communication. I recommend to specify this potential ambiguity from the beginning. Moreover, please note that no definition for the acronym IHI has been provided.

Response: We thank the reviewer for this helpful observation. We acknowledge that the acronym ISP may be confused with "ipsilateral silent period" (iSP), another established measure of transcallosal communication. We also note that the definition of IHI was previously missing. Both issues have been addressed in the revised manuscript.

Specifically, we have made the following changes:

1.Clarification of ISP ambiguity: In the Introduction, when ISP is first introduced, we have added the following clarification: " interhemispheric signal propagation (ISP; distinct from the ipsilateral silent period, another measure of transcallosal communication[5, 6]) " This explicitly distinguishes ISP from the similarly abbreviated iSP from the outset. Page 3, line 39-41

2.Definition of IHI: In the Discussion, where IHI first appears, we have now spelled out the full term: "interhemispheric inhibition (IHI)" to ensure clarity for readers unfamiliar with this abbreviation. Page13, line 300

4) The replication aim should be emphasized also in the title and/or in the abstract.

Response: We thank the reviewer for this suggestion. We have explicitly emphasized the replication aim in the abstract, as recommended.

Specifically, the title has been revised to reflect the independent replication nature of this study. The revised title is:

“Concurrent TMS-EEG to characterise interhemispheric signal propagation over the motor and prefrontal regions: an independent replication of GABAB receptor mechanisms”

Specifically, in the revised abstract, we state: " This study aimed to validate the sensitivity of a TMS-EEG-based measure of interhemispheric signal propagation (ISP) to GABAB receptor modulation, while independently replicating the pharmacological mechanisms of interhemispheric inhibition." Page 2, line 19-22

5) Methods:

a. Details on EMG recording are missing, which seems to be used at least for rMT calculation. Related to this, I am also wondering whether EMG was recorded also during TMS-EEG sessions, especially during motor cortex stimulation.

Response: We thank the reviewer for pointing out the missing details regarding EMG recording. During TMS-EEG recordings, EMG was not recorded, especially not during motor cortex stimulation. We have revised the “Resting motor threshold and TMS-EEG” subsection to address this concern.

Specifically, we have added the following information:“Resting motor threshold (RMT), defined as the minimum intensity to induce motor-evoked potentials (MEPs) > 0.05 mV of the first dorsal interosseous (FDI) muscle in 5/10 trials, was measured before each session. Surface electromyography (EMG) was recorded from the right FDI muscle using disposable Ag/AgCl electrodes in a belly-tendon montage during RMT determination only. During TMS-EEG recordings, EMG was not continuously monitored to avoid electrical interference with EEG signals and to minimize participant discomfort, consistent with established TMS-EEG protocols [23]. Single pulses to the hand region of the left M1 (45° to the midline, handle pointing backward) at 4s ± 10% jitter intervals were sent by a figure-eight coil connected to a Magstim Rapid2 system (Magstim Company Ltd, UK).” Page 6, line 113-122

b. Please specify whether the order was counterbalanced between experimental conditions.

Response: We thank the reviewer for this important methodological question. We have now explicitly clarified that the session order was randomized and counterbalanced.”

Specifically, in the “Experimental design and procedure” subsection, we have added the following text: “This study was a double-blind, placebo-controlled, crossover design with randomized and counterbalanced session order…. Specifically, participants were randomly allocated to one of two sequence orders (baclofen-placebo or placebo-baclofen) following a computer-generated randomization schedule (MATLAB, The MathWorks, USA). This procedure, conducted by an independent researcher not involved in data collection, ensured that potential sequence effects were evenly distributed between conditions[16, 17]” Page 5, line 87-93

c. The procedure for stimulation site localization should be described in greater detail, both for M1 and DLPFC. Further details should be provided also for noise masking procedure, as well as for coil orientation (please describe the direction of the current in the brain, e.g. anterior-to-posterior/posterior-to-anterior).

Response: We thank the reviewer for requesting additional details on stimulation site localisation, coil orientation, and noise masking. We have significantly expanded the “Resting motor threshold and TMS-EEG” subsection to address these points:“For M1 localization, the motor 'hotspot' was identified using a semi-grid systematic search procedure. The coil was initially positioned over the estimated hand knob area (approximately 5 cm lateral and 2 cm anterior to the vertex), then adjusted in 0.5-cm steps in an anterior-posterior and medial-lateral grid pattern to locate the site eliciting the largest peak-to-peak motor evoked potential (MEP) amplitude in the first dorsal interosseous (FDI) muscle. The identified hotspot was marked on the EEG cap for consistent repositioning across sessions. The coil was oriented with the handle pointing posterolaterally at a 45° angle relative to the sagittal midline, inducing a posterior-to-anterior (PA) current flow in the underlying cortex [24].For DLPFC localization, the left F3 position was determined using the Beam F3 methodology [25]. This approach measures the distance from nasion to inion and from left to right preauricular points, calculating F3 as the intersection point 50% of the distance from Fpz to FCz and 50% of the distance from F7 to Fz on the left side. The coil was oriented with the handle pointing laterally at a 90° angle relative to the sagittal midline (perpendicular to the midline), inducing a lateral-to-medial (LM) current flow [25, 26].” Page 6, line 126-Page 7,line 140

“During stimulation, white noise masking was delivered through inserted earphones at an intensity sufficient to render the TMS coil click barely audible[16].”Page 7, line 142-144

6) Analysis:

a. With a sampling rate of 5 kHz, the TMS artifact typically last about 5 ms (Veniero et al. 2009). Here, the authors reported an interpolation of 25 ms after the TMS pulse. Please provide the rationale of applying such a long time interpolation interval.

Response: We thank the reviewer for this critical methodological comment. We agree that the 25 ms interpolation window may remove early neural components such as P25/30. In the revised manuscript, we have provided a detailed rationale for this interpolation window and clarified that our ISP measure focuses on later time windows, which are not affected by this interpolation. We have added a detailed rationale in the “TMS-EEG data analysis” subsection of the revised Methods.

In the Methods section , we now state:“The large artifacts caused by the magnetic pulses were removed and interpolated between -5 and 25 ms. This interpolation window was chosen based on empirical evidence that TMS-related artifacts over lateral scalp regions (including motor and prefrontal cortices) can persist for up to 25-40 ms due to the activation of scalp muscles[30, 31]. Previous research has demonstrated that while amplifier recovery from the TMS pulse itself occurs within approximately 5-12 ms[32], a secondary artifact arising from cranial muscle activation requires a longer interpolation window for complete removal, particularly when stimulating lateral cortical areas[30]. This approach is consistent with established TMS-EEG preprocessing guidelines that recommend removing TMS-related artifacts before filtering and ICA decomposition to avoid contamination of neural signals[23]. Importantly, as our primary outcome measure (ISP) focuses on the 50-150 ms post-stimulus interval for the stimulated hemisphere and 60-160 ms for the contralateral hemisphere [7, 13, 33], the interpolation window does not affect the neural signals of interest.” Page 8, line 160- Page 8,line 173

In the Discussion section, we have additionally added the following clarification:

“Additionally, while our interpolation window of -5 to 25 ms is longer than the minimal amplifier recovery time of 5-12 ms reported in some studies[32], this conservative approach was adopted to ensure complete removal of secondary muscle artifacts that are particularly prominent when stimulating lateral cortical regions such as M1 and DLPFC[30, 31]. Rogasch et al. (2013) demonstrated that stimulation over lateral scalp positions produces a large secondary artifact that only fully recovers after approximately 25-40 ms, which is consistent with our interpolation window. Importantly, as our ISP measure focuses on the 50-160 ms post-stimulus window [7, 13], this interpolation does not compromise the neural signals central to our research question.” Page 15, line 347- Page 16 356

b. Please provide additional details on: how many components (range and average) were discarded in every steps; channel interpolation (type and how many channels were interpolated).

Response: We thank the reviewer for requesting additional details on ICA component rejection and channel interpolation. In the revised version, we have substantially expanded the “TMS-EEG data analysis” subsection to provide these missing details, including descriptive statistics for both ICA component rejection and channel interpolation.

For the first round of FastICA, we added:“On average, 10.0 ± 5.7 components were rejected for the motor cortex under the placebo condition, and 8.9 ± 4.1 under the baclofen condition. For the dorsolateral prefrontal cortex (DLPFC), 14.3 ± 5.5 components were rejected under placebo and 13.4 ± 5.3 under baclofen.” Page 8, line 176-179

For the second round of FastICA, we added:“For the motor cortex, an average of 25.9 ± 6.2 components were rejected under placebo and 25.0 ± 4.3 under baclofen. For the DLPFC, 19.1 ± 5.2 components were rejected under placebo and 18.4 ± 4.3 under baclofen. Blink, eye movement, muscle, and electrode noise components were identified and removed automatically based on predefined heuristics. All rejected components were visually inspected to ensure removal accuracy.” Page 9, line 181-186

For channel interpolation, we also provided descriptive statistics as follows: “Bad channels, if any, were interpolated using spherical spline interpolation.” Page 9, line 187

“The number of channels removed and interpolated was recorded for each condition. In the motor cortex, the mean number of interpolated channels was 1.00 ± 1.00 for baclofen and 0.89 ± 0.97 for placebo. In the dorsolateral prefrontal cortex, the mean number of removed channels was 8.74 ± 5.05 for baclofen and 7.52 ± 5.47 for placebo. No significant differences were observed between drug and placebo conditions in any region, indicating consistent data quality across sessions.” Page 9,line 188-193

c. Related to the previous points, a representative figure showing the averaged signal before and after the preprocessing would be highly informative (even in supplementary materials).

Response:

We thank the reviewer for this helpful suggestion. We agree that a representative figure showing the averaged signal before and after preprocessing would enhance the transparency of our preprocessing pipeline. In response to this comment, we have now included a supplementary figure (S1 Fig) in the revised manuscript.

Specifically, we have added the following sentence at the end of the TMS-EEG data analysis section：“A representative example of the EEG signal before and after preprocessing is provided in S1 Fig, illustrating the effectiveness of the artifact removal procedure across all conditions.” Page 9,line 200-202

d. It seems inaccurate to refer to the “cortex” when describing the ISP, considering that the analysis is at electrode-level without performing source reconstruction.

Response: We thank the reviewer for this important observation. We agree that referring to "cortex" when describing ISP based on electrode-level analysis is imprecise without source reconstruction. Following the reviewer's suggestion, we have revised the manuscript accordingly. Throughout the text, we have replaced "cortex" with more appropriate terms such as "region" (e.g., "motor region" and "prefrontal region") when referring to the electrode-level measurements, and used "hemisphere" (e.g., "stimulated hemisphere" and "unstimulated hemisphere") when describing the ISP calculation. These changes more accurately reflect the spatial resolution of our analysis. We believe this revision addresses the reviewer's concern and improves the precision of our terminology.

7) Results and interpretation

a. Was there any difference in rMT between conditions?

Response: We thank the reviewer for this important question. In the original manuscript, we did not report whether resting motor threshold (RMT) differed between conditions. In the revised manuscript, we have added a dedicated subsection in the Results section entitled “Resting motor threshold (RMT)” to address this point.

Specifically, we conducted a two-way repeated-measures ANOVA with factors Drug (baclofen vs. placebo) and Time (PRE vs. POST) on RMT values. Descriptive statistics are as follows:“A two-way repeated-measures ANOVA with factors Drug (baclofen vs. placebo) and Time (PRE vs. POST) was conducted on RMT values. Descriptive statistics showed that RMT was 64.00 ± 5.86 for baclofen PRE, 64.70 ± 5.70 for baclofen POST, 64.22 ± 6.12 for placebo PRE, and 65.19 ± 4.97 for placebo POST. The main effect of Time was significant (F(1,26) = 10.484, p = 0.003, partial η² = 0.287), indicating that RMT increased from PRE to POST regardless of drug condition. However, neither the main effect of Drug (F(1,26) = 0.588, p = 0.450, partial η² = 0.022) nor the Drug × Time interaction (F(1,26) = 0.321, p = 0.576, partial η² = 0.012) was significant. These results suggest that baclofen did not differentially affect RMT compared to placebo, and that the observed ISP effects are unlikely to be confounded by changes in stimulation intensity.”Page 11 , line 230-240

b. Among the limitations of the non-significant finding on DLPFC, a few methodological aspects may be mentioned, such as site localization and TMS intensity (due to differences between M1 in the cortical excitability of this area, scalp-to-cortex distances, optimal coil orientation, etc.)

Response: We thank the reviewer for this suggestion. We agree that several methodological factors may have contributed to the null finding in the dorsolateral prefrontal cortex (DLPFC). In the revised manuscript, we have added the following paragraph in the Discussion section to address this point: “Several methodological limitations may explain the null finding in the DLPFC. First, Beam F3 localization lacks MRI-guided precision, and individual anatomical variability may have resulted in suboptimal targeting. Second, 110% RMT intensity may be suboptimal for DLPFC given its greater scalp-to- region distance and different optimal coil orientation. These factors may reduce sensitivity to detect prefrontal drug effects.”Page 14, line 319-323

c. Other TMS measures have been described in the literature as index of transcallosal inhibition, such the ipsilateral silent period (e.g., Meyer et al., 1995) and measures derived from dual coil paradigms (e.g., Ferbert et al., 1992), as well as TMS-EEG measures, such as the M1-P15 TMS-evoked potential component (e.g., Bortoletto et al., 2021; Zazio et al., 2022). This literature should be incorporated in the introduction and/or discussion.

References

- Bortoletto M, Bonzano L, Zazio A, Ferrari C, Pedullà L, Gasparotti R, Miniussi C, Bove M. Asymmetric transcallosal conduction delay leads to finer bimanual coordination. Brain Stimul. 2021 Mar-Apr;14(2):379-388. doi: 10.1016/j.brs.2021.02.002. Epub 2021 Feb 10.

- Ferbert A, Priorit A, Rothwell JC, Day BL, Colebatchi JG, Marsden CD. Interhemispheric inhibition of the human motor cortex. J Physiol 1992.

- Meyer BU, Roricht S, Von Einsiedel HG, Kruggel F, Weindl A. Inhibitory and excitatory interhemispheric transfers between motor cortical areas in normal humans and patients with abnormalities of the corpus callosum. Brain

1995;118:429e40

- Veniero D, Bortoletto M, Miniussi C. TMS-EEG co-registration: on TMS-induced artifact. Clin Neurophysiol. 2009 Jul;120(7):1392-9. doi: 10.1016/j.clinph.2009.04.023. Epub 2009 Jun 16.

- Zazio A, Barchiesi G, Ferrari C, Marcantoni E, Bortoletto M. M1-P15 as a cortical marker for transcallosal inhibition: A preregistered TMS-EEG study. Front Hum Neurosci. 2022 Sep 16;16:937515. doi: 10.3389/fnhum.2022.937515.

Response: We thank the reviewer for this valuable suggestion. We agree that it is important to contextualize ISP within the broader literature on TMS-based measures of transcallosal communication. In the revised manuscript, we have added the following paragraph in the Introduction to address this point:“It is important to note that ISP represents one of several approaches to assess transcallosal inhibition. Other established measures include the ipsilateral silent period (iSP), which assesses transcallosal inhibition through suppression of ongoing EMG activity during voluntary contraction[6], and dual-coil TMS paradigms, which probe interhemispheric inhibitory circuits using conditioning and test pulses delivered to contralateral motor cortices [5]. More recently, TMS-EEG studies have identified the M1-P15 component as a putative marker of transcallosal inhibition, with shorter P15 latencies reflecting faster interhemispheric conduction[11, 12]. However, these measures are restricted to motor regions or require active muscle participation. In contrast, ISP can be applied to both motor and non-motor regions without requiring motor output, thereby extending the assessment of interhemispheric communication to prefrontal and other cortical areas [8-10].”Page 3 , line 44-54.

Reviewer #2: In this study, the authors using combined transcranial magnetic stimulation and electroencephalography (TMS-EEG) to probe alterations in interhemispheric signal propagation (ISP) across both the motor and prefrontal cortices following oral baclofen administration. Using a double-blind, placebo-controlled, crossover design, they report enhanced inhibition (reduced ISP) following baclofen, largely consistent with its known effects on GABAergic inhibition. This important study extends prior work assessing drug effects (including baclofen) on ISP recorded across motor and prefrontal regions using TMS-EEG; however, importantly it does so in a considerably larger sample. This research is important for understanding the neurochemical mechanisms involved, and helping to validate TMS-EEG as a method to explore these. Overall, this manuscript was clear, concise, and informative, and the methodology and analyses appear sound. I have some specific questions below that I hope the authors will be able to address.

1) I think a little more information on the chosen dose of baclofen (10 mg) would be beneficial, as it is not immediately clear why this does was selected. It is also different to the dose used in the prior Hui et al. 2019 study (50 mg).

Response: We thank the reviewer for this important question regarding the choice of baclofen dose (10 mg). We agree that the rationale for this dose selection should be clearly stated, especially given that it differs from the 50 mg dose used in the original Hui et al. (2020) study.

In the revised manuscript, we have added the following justification in the Methods: “The 10 mg dose was selected based on previous studies demonstrating that this dose produces measurable pharmacodynamic effects in healthy volunteers while maintaining good safety and tolerability profiles[18-20].This dose is consistent with prior TMS-EEG studies investigating GABAergic neurotransmission [21, 22].” Page 5, line 105-Page 6, line 108

In the Discussion section, we have added a new paragraph to further clarify the rationale for dose selection, including a comparison with the original study:“Regarding the choice of baclofen dose, we acknowledge that Hui et al. (2020) used a higher dose of 50 mg in their original TMS-EEG study. However, the 10 mg dose was selected in the present study for several reasons. First, 10 mg is the standard oral dose of baclofen used in human pharmacological studies in healthy volunteers, balancing safety, tolerability, and pharmacodynamic sensitivity. Second, previous TMS-EEG work has successfully demonstrated GABAB-mediated effects on TMS-evoked EEG potentials using the same 10 mg dose[21, 22]. Third, the primary goal of the present study was to validate ISP as a sensitive pharmacodynamic marker in a larger sample, which required a dose with established neurophysiological effects rather than the maximal tolerable dose.” Page 14 , line 324-Page 5,line 333

2) Can the authors please clarify how randomisation of session order (active, placebo) was determined? Similarly, I think some further justification on the use of the 90-minute waiting period after drug administration is needed – as no bloodwork was performed, it is important to be sure the analysis window captured peak (or close to peak) concentrations of the drug.

Response: We thank the reviewer for these important methodological questions regarding randomisation of session order and the justification for the 90-minute waiting period. We have addressed both points in the revised manuscript.

In the Methods, we have added the following clarification: “This study was a double-blind, placebo-controlled, crossover design with randomized and counterbalanced session order…. Specifically, participants were randomly allocated to one of two sequence orders (baclofen-placebo or placebo-baclofen) following a computer-generated randomization schedule (MATLAB, The MathWorks, USA). This procedure, conducted by an independent researcher not involved in data collection, ensured that potential sequence effects were evenly distributed between conditions[16, 17]” Page 5, line 87-93

We acknowledge that blood sampling was not performed to directly verify plasma concentrations. However, the 90-minute waiting period is well within the established peak concentration window (0.5–1.5 hours) for oral baclofen, and our significant ISP findings in the motor cortex provide indirect evidence that the drug was pharmacodynamically active at the time of post-drug assessments. We have noted the absence of blood sampling as a limitation in the Discussion: “Consistent with established protocols[21, 22], we implemented a 90-minute waiting period prior to post-drug TMS-EEG recordings. This duration was selected to ensure that TMS-EEG assessments coincided with peak or near-peak plasma baclofen concentrations, which typically occur 0.5–1.5 hours following oral administration, and to maintain consistency with the original study by Hui et al. (2020) that this study aims to replicate. Plasma concentration peaks were not systematically monitored due to the absence of blood sampling.”Page 15, line333-339

3) The authors mention some important limitations of the study, including the lack of MRI-guided neuronaviation for TMS-EEG and lack of blood sample collection. However, another important limitation is in regards to the potential for sensory components of the TMS stimulation (e.g., auditory and somatosensory) to lead to peripherally evoked potentials on the TMS-EEG record (e.g., see: Poorganji et al. [2023]. Scientific Reports, https://www.nature.com/articles/s41598-023-29920-2). While the authors did use noise masking, this does not always completely block out the sound of the TMS coil click. They do not mention using other methods such as foam between the coil and the scalp (to help dampen these responses), so I presume these were not performed? I think this should perhaps be noted as a limitation.

Response: We thank the reviewer for raising this important limitation regarding potential sensory confounds in TMS-EEG recordings. We agree that auditory and somatosensory inputs from TMS stimulation can evoke peripheral responses that may contaminate cortical signals. We did not use foam padding between the coil and scalp. In the revised manuscript, we have added the following paragraph in the Discussion section to address this point: “An additional limitation concerns potential sensory confounds in TMS-EEG recordings. TMS stimulation produces auditory and somatosensory inputs that can evoke peripheral responses contaminating cortical signals[49].Although we employed noise masking, this method does not eliminate auditory evoked potentials. We did not use additional control methods such as foam padding between the coil and scalp to dampen somatosensory responses. Consequently, the observed TEPs may reflect a mixture of direct cortical activation and sensory-evoked activity. However, the ratio-based ISP measure and the specificity of drug effects to the motor region (with null effects in DLPFC) argue against a purely sensory explanation…. Future studies should incorporate sham stimulation or sensory deprivation methods to further disentangle these contributions.” Page 16, line 358-375

4) I think it would be helpful if the authors were a little more clear in the abstract regarding the results. Specifically, they state “Our findings indicated that baclofen increased interhemispheric inhibition, indexed by decreased ISP. Moreover, this effect was more prominent over the motor cortex than the prefrontal cortex.” This could be interpreted as if there was an effect of baclofen on ISP in the prefrontal cortex, however, the ANOVA was not significant.

Response: We thank the reviewer for this helpful suggestion. We agree that the original phrasing — “this effect was more prominent over the motor cortex than the prefrontal cortex” — could be misinterpreted as implying a significant effect in the prefrontal cortex. In the revised manuscript, we have rewritten the abstract to accurately reflect the statistical findings.

The original sentence has been replaced with the following :“Our findings indicated that baclofen increased interhemispheric inhibition over the motor region, indexed by significantly decreased ISP (p = 0.037). No significant effect was observed in the dorsolateral prefrontal region (p = 0.186), suggesting regional specificity in GABAB receptor modulation of ISP.” Page 2, line 24-27

Minor

Page 6, line 90: I believe ‘EEP cap’ should be ‘EEG cap’.

Response: We thank the reviewer for catching this typographical error. We have corrected “EEP cap” to “EEG cap” in the revised manuscript. Page 6, line 124

Reviewer #3: The study by Zhou and colleagues examined how modulation of GABAB receptor activity influenced the response to TMS-evoked EEG potentials (TEP) generated by stimulation to dorsolateral prefrontal cortex (DLPFC) and primary motor cortex (M1). This was achieved by recording TEPs before and after oral intake of baclofen or placebo. The authors report a drug-related change in an index of interhemispheric communication. This paper is a replication of an older study, implemented with an increased sample size. While conceptually interesting, several elements of the methodology are not consistent with current best practice, which raises concerns about outcomes. In addition, several elements of the methods are unclear. Specific comments are listed below.

1. Stimulation appears to have been applied at 110% RMT for both sites. While this is fine for DLPFC, the suprathreshold intensity over M1 means that sensory reafference from motor evoked potentials would have confounded the TEP.

Response: We thank the reviewer for raising this important concern. We agree that suprathreshold M1 stimulation inevitably elicits MEPs, which may introduce proprioceptive and somatosensory reafference that could confound TEP recordings. In the revised manuscript, we have added a detailed discussion of this limitation and provided multiple lines of evidence to mitigate this concern.

The following statement has been added to the Methods section: “The choice of 110% RMT is consistent with established TMS-EEG protocols for M1 stimulation, as this suprathreshold intensity reliably elicits robust TEPs while maintaining tolerability[28, 29].”Page 7,145-147

The following paragraph has been added to the Discussion section :“Notably, the use of 110% RMT over M1 appears to be a common intensity in TMS-EEG research. A systematic review of 137 M1-TMS-EEG studies indicated that 74% of studies used suprathreshold stimulation intensities above resting motor threshold[28]. Furthermore, a study comparing 110% RMT, 90% RMT, and realistic sham stimulation suggested that EEG responses to suprathreshold M1 TMS primarily reflect direct cortical activation rather than sensory reafference, with re-afferent contributions accounting for approximately 10-20% of the total cortical response[29]. This finding is particularly relevant for our study, as the early TEP component P25 included in our ISP calculation has a short latency and may therefore be less affected by such re-afferent feedback. Additionally, our interhemispheric ratio approach, which calculates ISP as the ratio of contralateral to ipsilateral hemisphere activation, may help reduce bilateral sensory inputs. The drug-by-time interaction design also ensures that sensory conditions were comparable between baclofen and placebo sessions. The observation of a significant effect in the motor cortex but not in the dorsolateral prefrontal cortex suggests that a purely sensory explanation may be less plausible. Consistent with these considerations, our research group has previously applied 110% RMT over M1 in several TMS-EEG studies examining cortical excitability and interhemispheric communication[24, 50], supporting the feasibility of this parameter. ”Page 16 , line 375-Page 17, line 392

2. A total of 105 trials were collected, which would presumably mean that a lower number were included in the analysis. This is low for current standards. Descriptives of final trial numbers should be reported.

Response: We thank the reviewer for this important methodological concern. We agree that reporting the final number of trials retained after artifact rejection is essential for evaluating data quality. In the revised manuscript, we have added detailed descriptive statistics for trial numbers in the TMS-EEG data analysis section.

The following text has been added to the Methods section:“The number of trials retained after artifact rejection was recorded for each condition. In the motor cortex, the mean number of trials was 97.26 ± 2.84 for baclofen PRE, 97.04 ± 2.03 for baclofen POST, 96.89 ± 4.67 for placebo PRE, and 96.22 ± 2.34 for placebo POST. In the DLPFC, the mean number of trials was 83.33 ± 10.83 for baclofen PRE, 84.67 ± 8.16 for baclofen POST, 81.96 ± 12.97 for placebo PRE, and 81.89 ± 11.94 for placebo POST. These values indicate that a sufficient number of trials were available for reliable TEP estimation in all conditions.”Page 9, line193-200

3. Apologies if I missed it, but please specify the EEG amplifier that was used.

Response: We thank the reviewer for this clarification. In the revised manuscript, we have specified the EEG amplifier used in this study.

The “Resting motor threshold and TMS-EEG” subsection of the Methods now states: “Continuous EEG was recorded using a 64-channel cap with BrainAmp DC amplifiers (Brain Products GmbH, Germany) at a sampling rate of 5,000 Hz.”Page 7 , line 150-152

4. The TMS-related artifact peak was removed by excising data from -5 to 25 ms, relative to TMS application. The width of this window would have resulted in removal of neural activity associated with local cortical activation (i.e.,P25/30).

Response: We thank the reviewer for this critical methodological comment. We agree that the 25 ms interpolation window may remove early neural components such as P25/30. We have added a detailed rationale in the “TMS-EEG data analysis” subsection of the revised Methods.

In the Methods section, we now state: “The large artifacts caused by the magnetic pulses were removed and interpolated between -5 and 25 ms. This interpolation window was chosen based on empirical evidence that TMS-related artifacts over lateral scalp regions (including motor and prefrontal cortices) can persist for up to 25-40 ms due to the activation of scalp muscles[30, 31]. Previous research has demonstrated that while amplifier recovery from the TMS pulse itself occurs within approximately 5-12 ms[32], a secondary artifact arising from cranial muscle activation requires a longer interpolation window for complete removal, particularly when stimulating lateral cortical areas[30]. This approach is consistent with established TMS-EEG preprocessing guidelines that recommend removing TMS-related artifacts before filtering and ICA decomposition to avoid contamination of neural signals[23]. Importantly, as our primary outcome measure (ISP) focuses on the 50-150 ms post-stimulus interval for the stimulated hemisphere and 60-160 ms for the contralateral hemisphere [7, 13, 33], the interpolation window does not affect the neural signals of interest.” Page 8, line 160- Page 8,line 173

In the Discussion section, we have additionally added the following clarification:

“Additionally, while our interpolation window of -5 to 25 ms is longer than the minimal amplifier recovery time of 5-12 ms reported in some studies[32], this conservative approach was adopted to ensure complete removal of secondary muscle artifacts that are particularly prominent when stimulating lateral cortical regions such as M1 and DLPFC[30, 31]. Rogasch et al. (2013) demonstrated that stimulation over lateral scalp positions produces a large secondary artifact that only fully recovers after approximately 25-40 ms, which is consistent with our interpolation window. Importantly, as our ISP measure focuses on the 50-160 ms post-stimulus window [7, 13], this interpolation does not compromise the neural signals central to our research question.” Page 15, line 347- Page 16 356

5. The approach to the analysis is unclear. The methods section initially states that raw ISP values were compared between conditions and time points using rmANOVA. However, the results section then refers to calculation of change values that then appear to be the focus of the results. This needs to be clarified. In addition, details of how the change values were calculated, and how they were analysed statistically, should be provided. The plots in Fig 2C/3C appear to indicate that the analysis of change data included both Pre and Post timepoints (i.e., the Pre point is just 0), which isn’t appropriate.

Response: We thank the reviewer for pointing out this inconsistency in our analysis description. We have now clarified the analytical approach in the revised manuscript.

First, in the Statistical analysis section, our revision is as follows :“Statistical analyses were conducted using SPSS 25.0 (SPSS Inc., Chicago, IL, USA). For ISP, a two-way repeated-measures analysis of variance (rmANOVA) was performed on raw ISP values with drug condition (baclofen and placebo) and time point (PRE and POST) as within-subject factors. The critical test for drug effects was the Drug × Time interaction. For post-hoc comparisons, change scores (ΔISP = ISPPOST − ISPPRE) were calculated and compared between drug conditions using paired t-tests with Bonferroni correction (p < 0.05). These change scores were used for post-hoc analysis and visualization purposes only, and were not the dependent variable in the primary rmANOVA.”Page 10, line 216-224

Second, in the figure legends, our revisions are as follows: For Fig. 2C: “Changes in interhemispheric signal propagation (ISP%) following baclofen or placebo administration, calculated as POST minus PRE. The PRE time point is set to zero as a visual reference for 'no change from baseline', reflecting the mathematical definition of a change score (Δ = POST − PRE). Statistical analysis was performed on raw ISP values using repeated-measures ANOVA (see Methods). Baclofen significantly reduced ISP compared to placebo.” Page 11, line 249-Page 12, line 154

For Fig. 3C: “Changes in interhemispheric signal propagation (ISP%) following baclofen or placebo administration, calculated as POST minus PRE. The PRE time point is set to zero as a visual reference for 'no change from baseline', reflecting the mathematical definition of a change score (Δ = POST − PRE). Statistical analysis was performed on raw ISP values using repeated-measures ANOVA (see Methods). No significant difference was observed between baclofen and placebo.”Page 12, line262-267

6. The derivation of ISP values focusses on TEP data in the 50-150ms time window. This period is widely recognised within the current literature as likely reflecting indirect activity generated by sensory input (particularly auditory potentials). Consequently, it is highly likely that the reported results are confounded to some extent (possibly extensively) by sensory input.

Response: We thank the reviewer for raising this important concern. We agree that the 50-150 ms time window may be susceptible to sensory confounds. In the revised manuscript, we have added a detailed discussion of this limitation in the Discussion section.

In the Discussion section, we now state:“An additional limitation concerns potential sensory confounds in TMS-EEG recordings. TMS stimulation produces auditory and somatosensory inputs that can evoke peripheral responses contaminating cortical signals[49].Although we employed noise masking, this method does not eliminate auditory evoked potentials. We did not use additional control methods such as foam padding between the coil and scalp to dampen somatosensory responses. Consequently, the observed TEPs may reflect a mixture of direct cortical activation and sensory-evoked activity. However, the ratio-based ISP measure and the specificity of drug effects to the motor region (with null effects in DLPFC) argue against a purely sensory explanation…. Future studies should incorporate sham stimulation or sensory deprivation methods to further disentangle these contributions.” Page 16, line 358-375

7. Examination of butterfly plots shows that all conditions appear to contain responses that typify sensory contamination. Specifically, the signals are dominated by large N100-P180 complexes and have small, poorly differentiated early peaks. Examination of component topographies would help elucidate the source of these components, with fronto-central distributions providing further indication for the presence of sensory contamination. Given this, and without any control conditions, it remains difficult to identify the extent to which the reported data reflect direct vs indirect (sensory) brain responses.

Response: We thank the reviewer for this constructive suggestion. In response, we have added a supplementary figure (S2 Fig) showing the topographical voltage distribution for the peaks of interest.

We have also added the following to the Discussion: “S2 Fig illustrates the spatiotemporal evolution of voltage distribution over time, revealing that early components (P30, N45) exhibit localized distributions over the central-parietal region, in contrast to the fronto-central diffuse topography of N100-P180. This spatiotemporal differentiation suggests that these components may originate from distinct neural sources. While the fronto-central distribution of N100-P180 overlaps with auditory evoked potential topographies, the more localized, condition-specific patterns of early components support the interpretation that the ISP measure captures at least partially TMS-specific cortical activation rather than purely sensory contamination.” Page 16, line 366-374

8. Between-group comparisons of baseline TEPs do not appear to be provided. These should be reported to demonstrate data are not confounded by baseline differences in responses. Comparisons of other baseline characteristics should also be reported, including RMT and descriptives of analysis (i.e., number of epochs and ICs excluded).

Response: We thank the reviewer for this important methodological suggestion. In the revised manuscript, we have added several analyses to address baseline comparability between conditions.

First, for baseline TEP comparisons, we performed cluster-based permutation tests on TEP waveforms to compare baseline (PRE) responses between baclofen and placebo conditions. The following text has been added to the Discussion section: “To further investigate the effects of baclofen on local cortical excitability, we performed cluster-based permutation tests on TEP waveforms. In the M1 region, no significant differences were found between the baclofen and placebo groups across all predefined time windows (P25, N45, P60, N100, P180; all p > 0.05). In the DLPFC region, a significant positive cluster was detected only in the P60 time window (p = 0.036), indicating that TEP amplitudes in the baclofen group were significantly higher than those in the placebo group during this time window. No significant group differences were observed in the DLPFC for the other time windows (P25, N45, N100, P180; all p > 0.05). These results suggest that the effects of baclofen on local cortical excitability may be region- and time-specific.” Page 17, line 393-401

Second, for RMT baseline comparisons, we have added RMT analysis in the Results section: “In the revised manuscript, we have added a dedicated subsection in the Results section entitled “Resting motor threshold (RMT)” to address this point.

Specifically, we conducted a two-way repeated-measures ANOVA with factors Drug (baclofen vs. placebo) and Time (PRE vs. POST) on RMT values. Descriptive statistics are as follows:“A two-way repeated-measures ANOVA with factors Drug (baclofen vs. placebo) and Time (PRE vs. POST) was conducted on RMT values. Descriptive statistics showed that RMT was 64.00 ± 5.86 for baclofen PRE, 64.70 ± 5.70 for baclofen POST, 64.22 ± 6.12 for placebo PRE, and 65.19 ± 4.97 for placebo POST. The main effect of Time was significant (F(1,26) = 10.484, p = 0.003, partial η² = 0.287), indicating that RMT increased from PRE to POST regardless of drug condition. However, neither the main effect of Drug (F(1,26) = 0.588, p = 0.450, partial η² = 0.022) nor the Drug × Time interaction (F(1,26) = 0.321, p = 0.576, partial η² = 0.012) was significant. These results suggest that baclofen did not differentially affect RMT compared to placebo, and that the observed ISP effects are unlikely to be confounded by changes in stimulation intensity.” Page 11 , line 230-240

Third, for descriptive statistics of excluded ICs and epochs: We have added these details in the Methods section：For the first round of FastICA, we added:“On average, 10.0 ± 5.7 components were rejected for the motor cortex under the placebo condition, and 8.9 ± 4.1 under the baclofen condition. For the dorsolateral prefrontal cortex (DLPFC), 14.3 ± 5.5 components were rejected under placebo and 13.4 ± 5.3 under baclofen.” Page 8, line 176-179

For the second round of FastICA, we added: “For the motor cortex, an average of 25.9 ± 6.2 components were rejected under placebo and 25.0 ± 4.3 under baclofen. For the DLPFC, 19.1 ± 5.2 components were rejected under placebo and 18.4 ± 4.3 under baclofen. Blink, eye movement, muscle, and electrode noise components were identified and removed automatically based on predefined heuristics. All rejected components were visually inspected to ensure removal accuracy.” Page 9, line 181-186

For the number of trials retained after artifact rejection, we also provided descriptive statistics as follows:“The number of trials retained after artifact rejection was recorded for each condition. In the motor cortex, the mean number of trials was 97.26 ± 2.84 for baclofen PRE, 97.04 ± 2.03 for baclofen POST, 96.89 ± 4.67 for placebo PRE, and 96.22 ± 2.34 for placebo POST. In the DLPFC, the mean number of trials was 83.33 ± 10.83 for baclofen PRE, 84.67 ± 8.16 for baclofen POST, 81.96 ± 12.97 for placebo PRE, and 81.89 ± 11.94 for placebo POST. These values indicate that a sufficient number of trials were available for reliable TEP estimation in all conditions.” Page 9, line 193-202

9. It would be of interest to also investigate global changes in the TEP due to drug. Standard approaches would involve cluster-based comparisons targeting the early TEP potentials.

Response: We thank the reviewer for this excellent suggestion. In the revised manuscript, we have performed cluster-based permutation tests on TEP waveforms to investigate global changes in TEP due to baclofen.

Specifically, we added the following analysis: “However, within-group comparisons revealed no significant pre-post changes in the baclofen group for either the M1 or DLPFC regions across all predefined time windows (P25, N45, P60, N100, P180; all p > 0.05, cluster-corrected). This suggests that the observed group difference in the DLPFC P60 component may reflect baseline discrepancies between groups rather than a genuine drug effect. Alternatively, it could be driven by changes in the placebo group, such as practice effects or expectation effects.” Page 17, line 401-Page 18, line 407

In addition, we wish to notify the journal of an authorship change during this revision. Yiting He has been added as a co-first author, and Jiqing He has been added as a co-corresponding author, with written consent from all authors. A signed Authorship Change Form has been uploaded.

We hope that the revised manuscript is now suitable for publication in PLOS ONE. Sincerely,

Xianwei Che

Attachment Submitted filename: Response to reviewers.docx

# Peer review: Decision Letter 1

**DOI:** 10.1371/journal.pone.0360250.r003

**Authors:** Fengxue Qi

12 Jul 2026

Dear Dr. Che,

plosone@plos.org. When you're ready to submit your revision, log on to https://www.editorialmanager.com/pone/ and select the 'Submissions Needing Revision' folder to locate your manuscript file.

- A letter that responds to each point raised by the academic editor and reviewer(s). You should upload this letter as a separate file labeled 'Response to Reviewers'.
- A marked-up copy of your manuscript that highlights changes made to the original version. You should upload this as a separate file labeled 'Revised Manuscript with Track Changes'.
- An unmarked version of your revised paper without tracked changes. You should upload this as a separate file labeled 'Manuscript'.

If you would like to make changes to your financial disclosure, please include your updated statement in your cover letter. Guidelines for resubmitting your figure files are available below the reviewer comments at the end of this letter.

If applicable, we recommend that you deposit your laboratory protocols in protocols.io to enhance the reproducibility of your results. Protocols.io assigns your protocol its own identifier (DOI) so that it can be cited independently in the future. For instructions see: https://journals.plos.org/plosone/s/submission-guidelines#loc-laboratory-protocols. Additionally, PLOS ONE offers an option for publishing peer-reviewed Lab Protocol articles, which describe protocols hosted on protocols.io. Read more information on sharing protocols at https://plos.org/protocols?utm_medium=editorial-email&utm_source=authorletters&utm_campaign=protocols.

As the corresponding author, your ORCID iD is verified in the submission system and will appear in the published article. PLOS supports the use of ORCID, and we encourage all coauthors to register for an ORCID iD and use it as well. Please encourage your coauthors to verify their ORCID iD within the submission system before final acceptance, as unverified ORCID iDs will not appear in the published article. *Only*  the individual author can complete the verification step; PLOS staff *cannot*  verify ORCID iDs on behalf of authors.

We look forward to receiving your revised manuscript.

Kind regards,

Fengxue Qi, Ph.D.

Academic Editor

PLOS One

Journal Requirements:

If the reviewer comments include a recommendation to cite specific previously published works, please review and evaluate these publications to determine whether they are relevant and should be cited. There is no requirement to cite these works unless the editor has indicated otherwise.

[Note: HTML markup is below. Please do not edit.]

Reviewers' comments:

Reviewer's Responses to Questions

**Comments to the Author**

Reviewer #1: (No Response)

Reviewer #2: All comments have been addressed

Reviewer #3: (No Response)

**********

2. Is the manuscript technically sound, and do the data support the conclusions?

Reviewer #1: Yes

Reviewer #2: Yes

Reviewer #3: No

**********

3. Has the statistical analysis been performed appropriately and rigorously?

Reviewer #1: Yes

Reviewer #2: Yes

Reviewer #3: Yes

**********

4. Have the authors made all data underlying the findings in their manuscript fully available?

The PLOS Data policy

Reviewer #1: Yes

Reviewer #2: Yes

Reviewer #3: No

**********

5. Is the manuscript presented in an intelligible fashion and written in standard English?

Reviewer #1: Yes

Reviewer #2: Yes

Reviewer #3: Yes

**********

Reviewer #1: I greatly appreciate the authors’ efforts in revising the manuscript. The paper has improved substantially, and the authors have addressed all of the concerns raised in my previous review in a satisfactory manner.

I have no further major comments. I only have a few minor suggestions:

I suggest adapting the scale in Fig. S1 so that the EEG response to TMS (albeit largely artifactual) is visible and can be directly compared with the preprocessed response. As a reference, the authors may wish to consult Fig. S3 in the already cited paper by Zazio et al. (2022).

I suggest removing “consistent with established TMS-EEG protocols” (line 127), as well as the acronym iSP for ipsilateral silent period (line 51), as these may be misleading.

Reviewer #2: The authors have now adequately addressed my specific comments. I have no further questions for the authors.

Reviewer #3: 1. In response to my first comment, the authors refer to recent review that shows a suprathreshold intensity over M1 is common, and by extension acceptable. However, it’s prevalence in existing literature is not evidence that reflects good practice. Indeed, the review cited by the authors goes on to describe in detail why it is problematic (points that support my previous comment). As far as I can tell, the authors have in no way addressed this concern.

2. The authors state that ‘a sufficient number of trials’ were included in their TEP analysis, but do not provide any information on how they justify what they view as sufficient.

3. In response to my fourth comment, the authors refer to older literature to suggest that the stimulus artifact may last as long as 40ms. In the current state of the art, this should not be the case unless there is a problem with the recording. Despite this, the most effective approach for identifying the temporal window to interpolate would be to examine the length of the artifact in their own data.

4. The responses to my sixth and seventh comments only serve to further suggest that the data contributing to this paper are likely to be highly reflective of contamination from sensory artifacts.

5. Regarding the ICA process, it seems that a large number of components were removed, which would increase the possibility the neural activity of interest was unintentionally removed. In addition, the first ICA round removed on average between ~9 and 14 components. This round is only intended to remove large artifacts associated with the tail of TMS artifact, which generally is achieved by removing 1-2 components. Taken together, it seems that the ICA implementation may have been overly aggressive.

**********

what does this mean?). If published, this will include your full peer review and any attached files.

If you choose “no”, your identity will remain anonymous but your review may still be made public.

**Do you want your identity to be public for this peer review?**  For information about this choice, including consent withdrawal, please see our Privacy Policy

Reviewer #1: No

Reviewer #2: No

Reviewer #3: No

**********

[NOTE: If reviewer comments were submitted as an attachment file, they will be attached to this email and accessible via the submission site. Please log into your account, locate the manuscript record, and check for the action link "View Attachments". If this link does not appear, there are no attachment files.]

To ensure your figures meet our technical requirements, please review our figure guidelines: https://journals.plos.org/plosone/s/figures

You may also use PLOS’s free figure tool, NAAS, to help you prepare publication quality figures: https://journals.plos.org/plosone/s/figures#loc-tools-for-figure-preparation.

NAAS will assess whether your figures meet our technical requirements by comparing each figure against our figure specifications.

# Author comment: Author response to Decision Letter 2

**DOI:** 10.1371/journal.pone.0360250.r004

24 Aug 2026

Dear Editor and Reviewers,

We sincerely thank the Editor and the reviewers for their thoughtful and constructive comments on our manuscript (PONE-D-25-38064). These suggestions have greatly helped us improve the quality of our work. Our revisions are as follows.

Reviewer #1: I greatly appreciate the authors’ efforts in revising the manuscript. The paper has improved substantially, and the authors have addressed all of the concerns raised in my previous review in a satisfactory manner.

I have no further major comments. I only have a few minor suggestions:

1) I suggest adapting the scale in Fig. S1 so that the EEG response to TMS (albeit largely artifactual) is visible and can be directly compared with the preprocessed response. As a reference, the authors may wish to consult Fig. S3 in the already cited paper by Zazio et al. (2022).

2) I suggest removing “consistent with established TMS-EEG protocols” (line 127), as well as the acronym iSP for ipsilateral silent period (line 51), as these may be misleading.

Response: We thank the reviewer for the positive feedback and for these helpful suggestions. We have carefully addressed both points as follows:

Regarding Fig. S1 (now Fig. S3): We have adapted the scale of the figure to ensure that the raw EEG response to TMS is clearly visible and can be directly compared with the preprocessed response, following the reviewer's suggestion and the format of Fig. S3 in Zazio et al. (2022).

Fig. S3 Representative example of EEG signal before and after preprocessing.

The figure illustrates the effectiveness of the artifact removal procedure

across all conditions, showing averaged TEPs before preprocessing (upper panel)

and after preprocessing (lower panel) from a representative participant.

Regarding the suggested deletions: We have removed the phrase "consistent with established TMS-EEG protocols" from line 127(now line 119) in the Methods section. We have also removed the acronym "iSP" for ipsilateral silent period from line 51(now line 45) in the Introduction.

These changes have been made in the revised manuscript. We hope these revisions meet the reviewer's expectations

Reviewer #2: The authors have now adequately addressed my specific comments. I have no further questions for the authors.

Response: We sincerely thank the reviewer for the positive acknowledgment and for their valuable time and effort in reviewing our work.

Reviewer #3

1. In response to my first comment, the authors refer to recent review that shows a suprathreshold intensity over M1 is common, and by extension acceptable. However, it’s prevalence in existing literature is not evidence that reflects good practice. Indeed, the review cited by the authors goes on to describe in detail why it is problematic (points that support my previous comment). As far as I can tell, the authors have in no way addressed this concern.

Response: We agree with you that the available data cannot address this concern. We have now acknowledged this limitation.

“We acknowledge that the use of suprathreshold stimulation intensity (110% RMT) over M1 means that sensory reafference from motor evoked potentials may have confounded the TEP. It is noted that our ISP measure is calculated as a ratio of contralateral to ipsilateral hemisphere activation, which may reduce bilateral sensory contributions, as sensory reafference would affect both hemispheres. However, this indirect consideration does not substitute for direct experimental controls. Future studies should incorporate more sophisticated sensory deprivation methods to further disentangle the contributions of direct cortical activation and sensory-evoked activity.” Page 17, Line 390-397

2. The authors state that ‘a sufficient number of trials’ were included in their TEP analysis, but do not provide any information on how they justify what they view as sufficient.

Response: Thank you for this critical methodological point. To justify the “sufficient number of trials” claim, we now provide literature-based evidence in the revised Methods section.

“To justify the sufficient number of trials, established TMS-EEG literature was referred. For the M1, Lioumis et al. (2009) reported a high reproducibility (r > 0.80) with ~100 pulses per condition and at least 80 epochs retained for averaging [35]. For the DLPFC, Noda et al. (2024) demonstrated that 80 pulses yield TEP waveforms equivalent to 160 or 240 pulses, while 40 pulses are insufficient [36]. In our dataset, the retained trial numbers are sufficient for reliable TEP estimation.” Page 9, Line 199- Page 10, Line 205

3. In response to my fourth comment, the authors refer to older literature to suggest that the stimulus artifact may last as long as 40ms. In the current state of the art, this should not be the case unless there is a problem with the recording. Despite this, the most effective approach for identifying the temporal window to interpolate would be to examine the length of the artifact in their own data.

Response: We thank the reviewer for this important methodological comment. We have now examined the artifact duration in our own data.

“The -5 to 25 ms interpolation window was selected based on direct inspection of the TMS-related artifact in this dataset. While the initial amplifier recovery occurred within approximately 5–12 ms, a secondary artifact component arising from cranial muscle activation was observed to persist up to approximately 20–25 ms, with considerable inter-individual variability in its duration. To ensure complete and consistent removal of the full artifact time course for all participants, a conservative fixed window of -5 to 25 ms was applied. A representative example of the artifact and the interpolation window is provided in Fig. S1.” Page 8, Line 162-169

Fig. S1 The figure shows the averaged TMS-EEG signal (electrode Fz) from a representative participant under DLPFC stimulation.

4. The responses to my sixth and seventh comments only serve to further suggest that the data contributing to this paper are likely to be highly reflective of contamination from sensory artifacts.

Response: We thank the reviewer for this critical observation. We fully acknowledge that sensory confounds represent a major challenge in TMS-EEG research. In our revised manuscript, we have now taken additional steps to empirically address this concern. We have now examined the influence of Cz, which is sensitive to global EEG fluctuations and non-specific sensory inputs, on our main findings.

“To control for potential confounding effects of global brain activity and sensory artifacts, we examined Cz butterfly plot and performed an additional control analysis in which the change score (POST − PRE) of Cz was entered as a covariate in the repeated-measures ANOVA. The Cz electrode, located at the midline vertex, is sensitive to global EEG fluctuations and non-specific sensory inputs, and was therefore selected a priori as an index of global cortical activity. The butterfly plot showed that Cz had a clear 100-200 ms complex close to the sensory artefacts (Fig. S4). Statistical analyses indicated that Cz covariate did not significantly predict ISP changes (F(1,51) = 1.138, p = 0.291). Critically, the time × group interaction on ISP remained significant after controlling for Cz activity (F(1,51) = 4.126, p = 0.047, partial η² = 0.075), indicating that the baclofen-induced reduction in ISP cannot be fully accounted for by global EEG changes or non-specific sensory influences.” Page 12, Line 261-271

Fig. S4 Butterfly plots of TEPs at the Cz electrode before and after drug administration. (A) Pre-baclofen and (B) post-baclofen TEP butterfly plots; (C) pre-placebo and (D) post-placebo TEP butterfly plots. The Cz electrode is highlighted in purple, and the vertical gray bar indicates the timing of the TMS pulse.

Response: We have also acknowledged the importance of sensory deprivation methods in future studies.

“Future studies should incorporate more sophisticated sensory deprivation methods to further disentangle the contributions of direct cortical activation and sensory-evoked activity.” Page 17, Line 395-397

5. Regarding the ICA process, it seems that a large number of components were removed, which would increase the possibility the neural activity of interest was unintentionally removed. In addition, the first ICA round removed on average between ~9 and 14 components. This round is only intended to remove large artifacts associated with the tail of TMS artifact, which generally is achieved by removing 1-2 components. Taken together, it seems that the ICA implementation may have been overly aggressive.

Response: We thank the reviewer for raising this important methodological concern. In our pipeline, we manually inspected every single component marked for removal at each ICA round to ensure that only artifactual components were removed while neural signals were preserved. To illustrate this, we have now included a supplementary figure (Fig. S2) in the revised manuscript, showing an example of the manual inspection performed during the first ICA round. As shown in the figure, the components identified for removal exhibited characteristic features of TMS-related artifacts (e.g., sharp waveforms, focal scalp topographies, and spectral profiles consistent with known artifact patterns). This manual inspection procedure was applied to all participants and all conditions, ensuring the accuracy of artifact rejection while minimizing the risk of inadvertently removing neural activity. We have now added the following clarification in the Methods section.

“The number of components removed in the first ICA round reflects the cumulative total of artifactual components identified across multiple ICA runs, with each step manually inspected to confirm that only artifactual components were removed while neural signals were retained (Fig. S2).” Page 8, Line 178- Page 9, Line 181

Fig. S2 Representative example of manual component inspection during the first ICA round. The figure shows a comparison of artifactual components identified for removal and neural components retained during the first ICA round for a representative participant, including component waveforms, scalp topographies, and power spectra. The removed components exhibit characteristic features of TMS-related artifacts (e.g., sharp waveforms, focal scalp distributions, and spectral profiles consistent with known artifact patterns), whereas the retained components preserve typical TEP waveform features. Similar manual visual inspections were performed for all participants and all conditions.

Response: We have also discussed the possibility of an overly aggressive first round of ICA.

“In addition, the number of components removed in the first ICA round reflects the cumulative total of artifactual components (~9-14 components). There is a possibility that it may have been overly aggressive for a first round of ICA.” Page 18, Line 412-414

Overall, we really appreciate the reviewers for providing these great suggestions. This work is now much improved. We wish these changes are satisfactory.

Attachment Submitted filename: Response_to_reviewers_auresp_2.docx

# Peer review: Decision Letter 2

**DOI:** 10.1371/journal.pone.0360250.r005

**Authors:** Fengxue Qi

25 Sep 2026

Concurrent TMS-EEG to characterise interhemispheric signal propagation over the motor and prefrontal regions: an independent replication of GABAB receptor mechanisms

PONE-D-25-38064R2

Dear Dr. Che,

We’re pleased to inform you that your manuscript has been judged scientifically suitable for publication and will be formally accepted for publication once it meets all outstanding technical requirements.

Within one week, you’ll receive an e-mail detailing the required amendments. When these have been addressed, you’ll receive a formal acceptance letter and your manuscript will be scheduled for publication.

An invoice will be generated when your article is formally accepted. Please note, if your institution has a publishing partnership with PLOS and your article meets the relevant criteria, all or part of your publication costs will be covered. Please make sure your user information is up-to-date by logging into Editorial Manager at Editorial Manager® and clicking the ‘Update My Information' link at the top of the page. For questions related to billing, please contact billing support.

If your institution or institutions have a press office, please notify them about your upcoming paper to help maximize its impact. If they’ll be preparing press materials, please inform our press team as soon as possible -- no later than 48 hours after receiving the formal acceptance. Your manuscript will remain under strict press embargo until 2 pm Eastern Time on the date of publication. For more information, please contact onepress@plos.org.

Kind regards,

Fengxue Qi, Ph.D.

Academic Editor

PLOS One

Reviewers' comments:

Reviewer's Responses to Questions

**Comments to the Author**

Reviewer #1: All comments have been addressed

**********

2. Is the manuscript technically sound, and do the data support the conclusions?

Reviewer #1: (No Response)

**********

3. Has the statistical analysis been performed appropriately and rigorously?

Reviewer #1: (No Response)

**********

4. Have the authors made all data underlying the findings in their manuscript fully available?

The PLOS Data policy

Reviewer #1: (No Response)

**********

5. Is the manuscript presented in an intelligible fashion and written in standard English?

Reviewer #1: (No Response)

**********

Reviewer #1: (No Response)

**********

what does this mean?). If published, this will include your full peer review and any attached files.

If you choose “no”, your identity will remain anonymous but your review may still be made public.

**Do you want your identity to be public for this peer review?**  For information about this choice, including consent withdrawal, please see our Privacy Policy

Reviewer #1: No

**********

# Editor report: Acceptance letter

**DOI:** 10.1371/journal.pone.0360250.r006

**Authors:** Fengxue Qi

PONE-D-25-38064R2

PLOS One

Dear Dr. Che,

I'm pleased to inform you that your manuscript has been deemed suitable for publication in PLOS One. Congratulations! Your manuscript is now being handed over to our production team.

At this stage, our production department will prepare your paper for publication. This includes ensuring the following:

* All references, tables, and figures are properly cited

* All relevant supporting information is included in the manuscript submission,

* There are no issues that prevent the paper from being properly typeset

You will receive further instructions from the production team, including instructions on how to review your proof when it is ready. Please keep in mind that we are working through a large volume of accepted articles, so please give us a few days to review your paper and let you know the next and final steps.

Lastly, if your institution or institutions have a press office, please let them know about your upcoming paper now to help maximize its impact. If they'll be preparing press materials, please inform our press team within the next 48 hours. Your manuscript will remain under strict press embargo until 2 pm Eastern Time on the date of publication. For more information, please contact onepress@plos.org.

You will receive an invoice from PLOS for your publication fee after your manuscript has reached the completed accept phase. If you receive an email requesting payment before acceptance or for any other service, this may be a phishing scheme. Learn how to identify phishing emails and protect your accounts at https://explore.plos.org/phishing.

If we can help with anything else, please email us at customercare@plos.org.

Thank you for submitting your work to PLOS One and supporting open access.

Kind regards,

PLOS One Editorial Office Staff

on behalf of

Assoc. Prof. Dr. Fengxue Qi

Academic Editor

PLOS One
